# Reproducing scientists’ mobility: a data-driven model

**DOI:** 10.1038/s41598-021-90281-9

**Published:** 2021-05-24

**Authors:** Giacomo Vaccario, Luca Verginer, Frank Schweitzer

**Affiliations:** 1 Chair of Systems Design, ETH Zürich, 8092 Zurich, Switzerland; 2 AXES Lab, IMT School for Advanced Studies Lucca, 55100 Lucca, Italy

**Keywords:** Physics, Applied physics

## Abstract

High skill labour is an important factor underpinning the competitive advantage of modern economies. Therefore, attracting and retaining scientists has become a major concern for migration policy. In this work, we study the migration of scientists on a global scale, by combining two large data sets covering the publications of 3.5 million scientists over 60 years. We analyse their geographical distances moved for a new affiliation and their age when moving, this way reconstructing their geographical “career paths”. These paths are used to derive the world network of scientists’ mobility between cities and to analyse its topological properties. We further develop and calibrate an agent-based model, such that it reproduces the empirical findings both at the level of scientists and of the global network. Our model takes into account that the academic hiring process is largely demand-driven and demonstrates that the probability of scientists to relocate decreases both with age and with distance. Our results allow interpreting the model assumptions as micro-based decision rules that can explain the observed mobility patterns of scientists.

## Introduction

Scientists are highly mobile individuals. This has been true in the past and is even more true today^1^. A thorough understanding of the relocation patterns and determinants is valuable for national research and immigration policy to respond and appreciate the mechanisms behind global scientists’ mobility. According to the OECD^2^, this mobility is a “key driver of knowledge circulation worldwide”, with implications for the competitive advantage of advanced knowledge economies. Therefore, an increasing number of works analyses the mobility of scientists and their motivation to relocate^3,4^. Many publications have focused on the relationship between movements and scientific impact^5–10^. Other works have analysed scientists mobility within and across *countries*, to determine policy impacts^11,12^ or to study the brain circulation phenomenon^13–16^.

While most of these studies focus on the aggregated level, e.g., on bilateral flows between countries, there is a need to better understand scientific mobility at the individual level^17,18^. Empirical works in this direction^19,20^ are often based on survey data that provide only partial coverage of the global mobility of scientists. Theoretical works on scientist mobility^21^, on the other hand, are rarely validated against data. Researches in complexity and network theory have mostly analyzed scientific collaborations^22–24^ or hiring practices^25^.

Our work addresses this research gap in a two-fold manner. First, we provide empirical insights into scientific mobility at the individual level, by reconstructing 3.5 million geographical career paths from large-scale data sets. Second, we provide an agent-based model that is calibrated against the available data and is capable of reproducing the distributions of relocation *distances* and relocation *age*. In developing our *data-driven* model, we follow the approach of^26–28^. Our model incorporates two factors that are known to affect academic mobility: (i) geographical distance^4,29^, (ii) prestige, or selectiveness of academic institutions^25,30,31^. We also contribute to the understanding of global mobility, by reconstructing and analysing the world network of scientists mobility at the level of *cities*, not countries. From this global network, we extract topological features such as the distributions of degrees, local clustering coefficients, path lengths, and assortativity, to demonstrate that these can also be reproduced by our agent-based model.

With the present study, we provide a parsimonious baseline model that replicates many defining features solely relying on geographic distance and “scientific impact” measures in addition to the interaction rules describing a simplified academic labor market. This model might serve as a starting point for more complex refinements taking into account many more factors we know to be important in the relocation choices of high-skill labour, e.g., city amenities^4^, national borders and language^16^. Nevertheless, by *only* considering two factors, scientific impact and geography, the model highlights their fundamental role in understanding scientists’ mobility.

## Results

### Empirical findings

By combining two large-scale bibliographic datasets as described in “Materials and methods” section we obtain for $N= 3,740,187$ scientists information about the sequence of cities they worked in their careers, between 1950 and 2009 from PubMed. The merged dataset contains $M=5485$ unique cities. The data allows us to construct the geographical “career path” of these scientists. An illustrative example is given in Table S1. In short the geographical career paths are the sequence of locations an author has had an affiliation with. Note that a move corresponds to a relocation from a city to different city (i.e., mobility within a city is not considered a move).

#### Statistics of geographical career paths

From the career paths we compute the *relocation distances* scientists moved when changing their affiliation, using the Haversine formula for geodesic distances.

**Figure 1:**
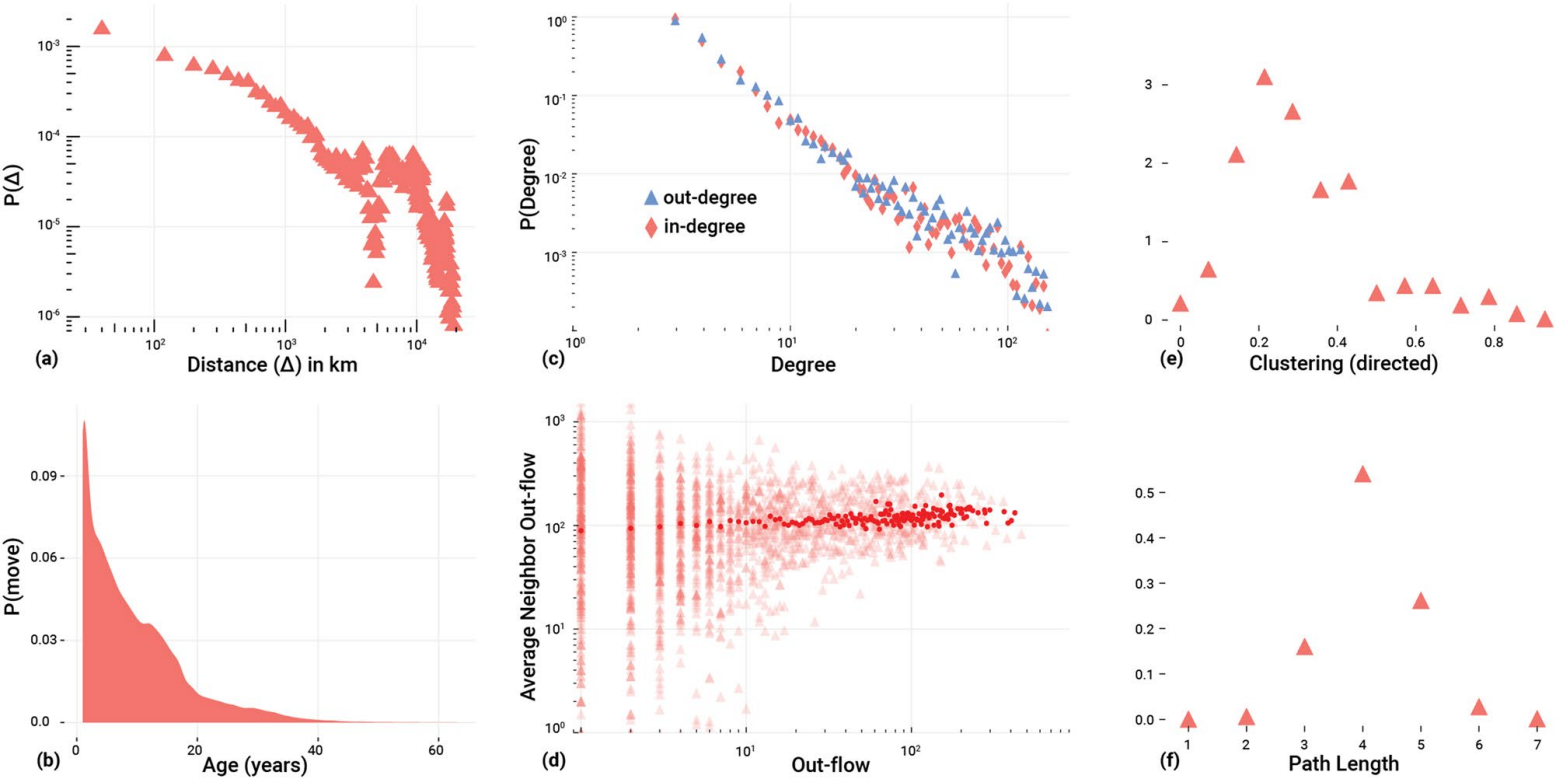
Characterization of the empirical academic mobility. At the individual level, we have the distribution of relocation distances of scientists (**a**) and the distribution of moves dependent on the (academic) age of scientists (**b**). At the global level, we reconstruct the mobility network for which we plot the distribution of degrees (**c**), path lengths (**f**), and local clustering coefficients (**e**). In (**d**), we plot the average out-flow of neighbors of a city a function of its out-flow: each red triangles represents a city, while the red points represent averages taken over cities with the same out-flow. Note that the plots in (**a**,**c**,**d**) are in log-log.

The distribution obtained from 62,465 scientists relocating between 2000 and 2008 is shown in Fig. 1a. We note that it is a left-skew distribution with a median of 1000 km, i.e., most scientists find a new affiliation within a radius of 1000 km around their current affiliation. However, relocations toward cities that are more than 5000 km away are also quite frequent.

The data also allows us to relate the frequency of such moves to the age of scientists. Because the physical age of scientists is unavailable, we rely on their *academic age*, $t^{a}_{i}$, also measured in years. $t_{i}^{a}=0$ when the scientist publishes his/her first paper, according to our database. The frequency of any recorded moves over the academic age $t^{a}$ is shown in Fig. 1b. Again, it is a left-skew distribution with a median of 7 years. This matches the known fact that the mobility of scientists drastically decreases with age^32,33^. However, we also identify that some scientists change their working location after been active for 40 years.

#### Reconstructing the mobility network of scientists

While the career paths and their statistics refer to individual scientists, we can also analyse the network that results from aggregating all of the career paths of a given year. This aggregation changes the unit of analysis to the *city level*. For each year, we obtain the number of scientists $N_{K}(t)$ in a given city *K* from their publications by taking unique geo-located scientists into account.

**Figure 2:**
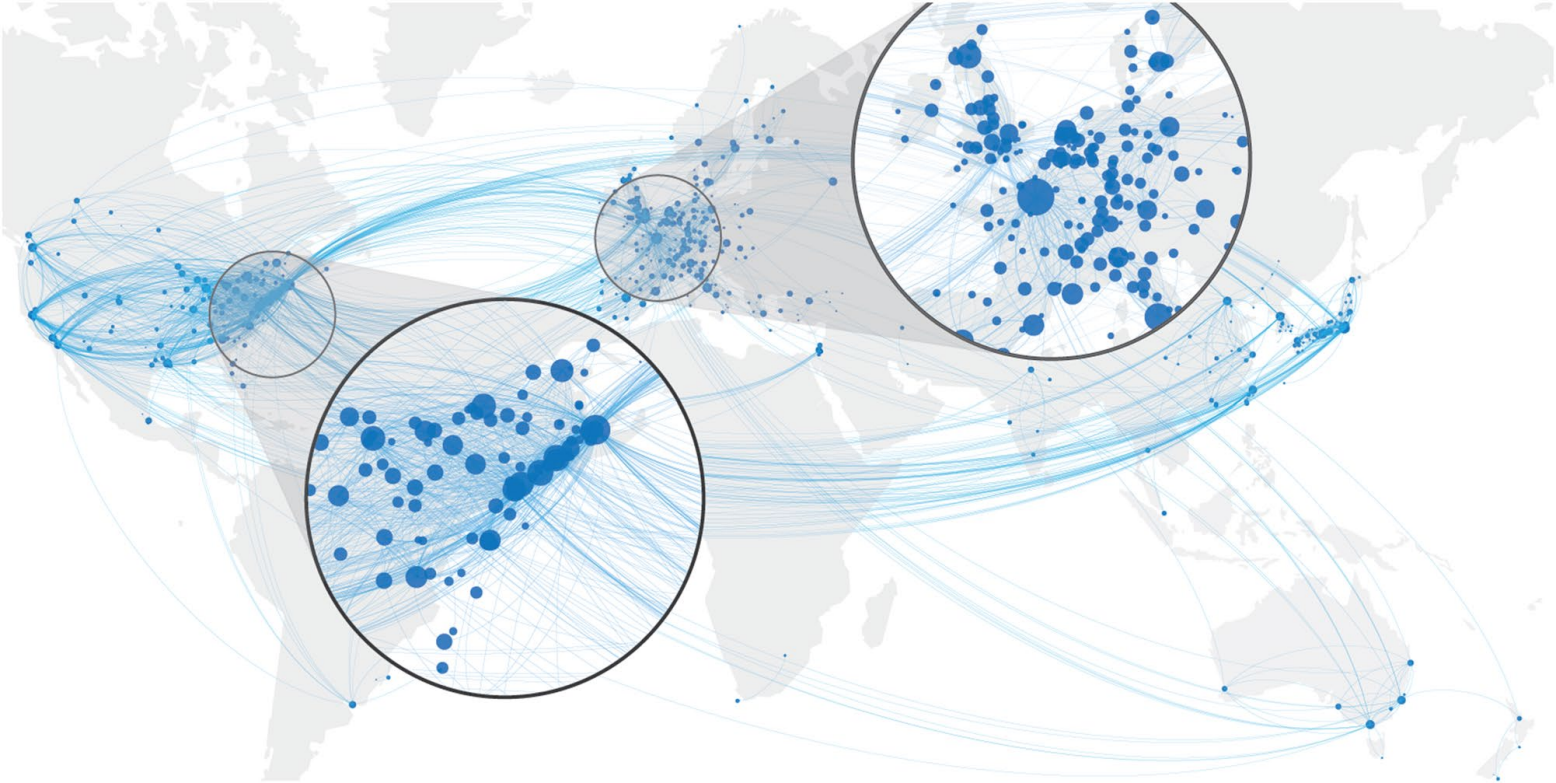
The intercity mobility network at global scale. Only locations with at least 50 active scientists in the period 2000 to 2006 are shown. The link width indicates the logarithm of the flows between the given cities. The node size is proportional to the number of scientists stationary in that city.

We further calculated for each year *t* the number of scientists $\Delta N_{K\leftarrow L}(t)$ moving into city *K* from another city *L*, i.e., the inflow, and the number of scientists $\Delta N_{L\leftarrow K}(t)$ moving out of city *K* to another city *L*, i.e. the outflow. For any given pair (*K*, *L*) of cities, we then calculate the *total flow* of scientists between these two cities, $\Delta N_{L\leftarrow K} + \Delta N_{K\leftarrow L}$. These flows allow us to visualise the mobility network of scientists at the world level, shown in Fig. 2. The links are directed and weighted according to the total flow.

#### Topological properties of the mobility network

To obtain the topological properties common in network analysis, we aggregate the mobility networks for the period 2000–2008. On this *aggregated network*, we calculate standard measures, such as the *in-degree* and the *out-degree distributions* where the in-degree of a city is the number of distinct cities from where scientists arrive; while the out-degree of a city is the number of distinct cities to where scientists leave. Figure 1c shows that these are a broad distributions. Some cities act as hubs, with a large degree, most cities, however, only have a small degree.

The distribution of *path lengths*, shown in Fig. 1f, measures the minimum number of steps needed to reach city *i* from another city *j* on the directed network. The small number of steps indicates that the network is dense in a *topological sense*, not necessarily in a geographical one.

The *local clustering coefficient*, on the other hand, measures whether three neighbouring cities (with respect to their geographical proximity) form closed triangles, i.e., whether there is an exchange of scientists among all three of them. Figure 1e shows the distributions of these values, and we find that most cities have a small local clustering coefficient.

The *out-neighbor connectivity*, eventually, measures to what extent cities with a certain out-flow (i.e., weighted out-degree) are connected to other cities with a similar out-flow. Figure 1d shows that for cities with low out-flow there is a higher dispersion, i.e., these are connected to both cities with high and low out-flow. Whereas there cities with high out-flow are on average connected to other cities with high out-flow. Even though this would suggest a positive assortativity, the mobility network has a neutral (or slightly negative) assortativity coefficient (see Table S6 in the SI).

### Modeling the mobility of scientists

We now develop a model to reproduce the characteristic empirical properties of the scientists’ mobility network discussed above. Precisely, we want to reproduce features both at *scientist* and *network* level. These are, at the scientist level, (i) the distribution of moved distances, Fig. 1a and (ii) the “age at move” distributions, Fig. 1b. At the network level, we want to reproduce (iii) the distributions of the topological features shown in Fig. 1c–f, i.e., degrees, local clustering coefficients, path lengths, and average neighbour degree.

We develop an *agent-based model* because we want to model the relocation of *scientists*, as opposed to a system dynamics model that would merely reproduce the flows between *cities*. This choice implies that *macroscopic features* describing the system, such as the topological properties already discussed, must be *emergent properties* arising from the agent dynamics.

Our model is composed of two entities, agents and locations. Agents represent scientists. Each agent *i* is characterized by three properties that change over time: its position, ${r}_i(t)$, its fitness, $f_i(t)$, and its years of activity $y_i(t)$. Time is measured in discrete simulation steps, each step representing 1 year. When we start our simulations at time $t=0$, which is chosen as the year 2000 below, we have to consider that many agents have already published before 2000, which is included in $y_{i}(t)$. For instance, an agent that published its first paper in 1995 will have a $y_{i}(2000)=5$. This information is essential to measure an agent’s fitness, $f_{i}(t=0)$, which we do below.

Locations represent cities and host agents. Each location *K* is characterised by three properties that can also partly change over time: its position ${R}_{K}$ defined in real geographical space by means of longitude and latitude, its fitness, $F_{K}(t)$, and the number of agents it hosts, $N_{K}(t)$. Note that ${R}_{K}$ and $N_{K}(t)$ are taken from the available empirical data. For the position ${r}_i(t)$ of an agent, we assume that at each time step the agent can be found in one of the available locations. So ${r}_i(t)=R_{K}$ where *K* is the location, where agent *i* is located at time *t*.

#### Agent and location fitness

The individual agent fitness $f_i(t)$ represents the academic impact of a scientist. We proxy this impact by the papers that he/she has co-authored. Precisely, we assign to each paper a score equal to the impact factor of the journal (taken from SCImago) where it was published divided by the number of co-authors. Then, for each scientist, we aggregate the scores of his/her co-authored papers in the last 2 years of activity. By this, we obtain a distribution of fitness values that we can assign to agents.

We assign to each location *K* a fitness value $F_{K}(t)$ reflecting the quality of the academic institutions hosted in a city. To make this idea explicit and measurable, we take $F_{K}(t)$ to be the mean fitness of the agents located in *K*. Note that this approach is in line with how rankings of academic institutions are created. Indeed, university rankings are determined considering the academic impact and quality of the scientists working there. In our model, we assume that the $F_{K}(t)$ is public information, and thus, agents may use this information in their decision rule.

#### Relocation preferences

Our central modelling assumption is that agents prefer to work in locations that provide a higher fitness than the one they are currently based. This assumption is rooted in the finding that there is positive selection of prolific scientists to move to larger and more connected cities^31^. These locations, however, can be distant from the current location, which implies higher relocation costs. Therefore, an agent *i* takes into account the fitness $F_{K}(t)$ of locations and its geodesic distance $\Delta _{i, K}(t)$. Agents combine this information in a *re-scaled fitness score* $\tilde{F}_{i,K}(t) = F_{K}(t) / (\Delta _{i, K}(t))^b$ for each location *K*. *b* is a model parameter, used to weight the impact of spatial distances. The bigger *b*, the more important any spatial distance becomes.

Ranking the values $\tilde{F}_{i, K}(t)$ from high to low, each agent then obtains an *individual* ranking that reflects its preferences where to move to. Agents in *L* will consider only those locations where ${F}_{K}(t)>F_{L}(t)$, i.e., where the average fitness of scientists is larger than the average fitness of scientists in their city. Hence, each agent *i* assigns to a location *K* the score:

1 $$\begin{aligned} R(i,K) = \Theta \left[ {F}_{K}(t) - F_{L}(t)\right] \frac{F_{K}(t)}{(\Delta _{i, K}(t))^b}, \end{aligned}$$

where $\Theta \left[ {F}_{K}(t) - F_{L}(t)\right]$ is equal to 1 when ${F}_{K}(t)>F_{L}(t)$ and equal to 0, otherwise.

#### Relocation decisions

Agents only come up with a ranked list of possible locations they would consider to move to (and we assume that they send applications to the academic institutions in these locations). However, agents do not decide where to move. This decision, whether or not to accept the agent, is taken by the location.

A location *K* will accept new agents only if it has sufficient capacity. The capacity $N_{K}(t)$ for a given city *K*, is estimated from the number of scientists empirically observed in city *K* in year *t*. Dependent on the individual ranking of agents, some locations obtain more applications than the capacity allows them to accept. So each location ranks the qualified agents according to their fitness $f_{i}(t)$. Available slots are filled starting from agents with higher fitness values until the capacity $N_{K}(t)$ is reached. Precisely, if $f_i(t)>F_K(t)$, location *K* considers agent *i* with probability $p=1$ because this allows location *K* to increase its fitness $F_{K}(t)$. If $f_i(t)\le F_K(t)$, location *K* considers agent *i* only with a probability $p = (f_i(t)/F_{K})^s$ where *s* is our second model parameter. This parameter *s* represents the *selectiveness* of locations, the higher *s*, the more difficult it is to be hired. Hence, if a location *K* has some openings, its probability to accept agent *i* is:

2 $$\begin{aligned} p(K,i) = {\left\{ \begin{array}{ll}1 &{} f_i(t) > F_{K}(t)\\ \left( \frac{f_i(t)}{F_{K}(t)}\right) ^{s} &{} \text {otherwise} \end{array}\right. }. \end{aligned}$$

**Figure 3:**
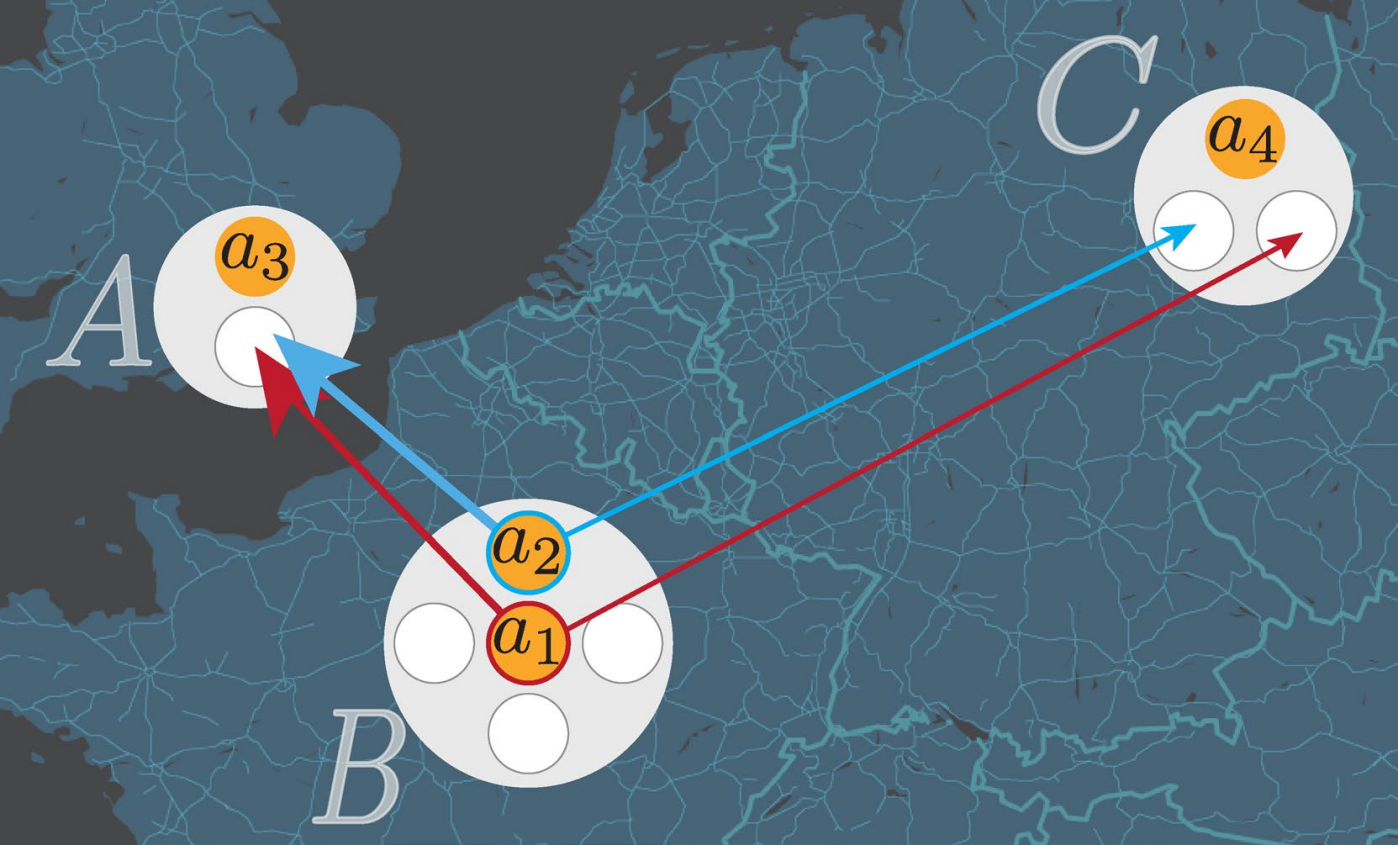
Example of relocation rules. Four agents ($a_1$, $a_2$, $a_3$ and $a_4$) are hosted in three locations, *A*, *B* or *C*, representing London, Paris and Berlin. Each location has a maximum number of available positions illustrated by small circles: $N_A = 2$, $N_B = 5$ and $N_C = 3$. In this image, agents $a_1$ and $a_2$ compute the rescaled fitness of the available locations (*A* and *C*) and rank these location accordingly. Here, we have assumed that *A* and *C* have the same fitness ($F_A(t) = F_C(t)$), but *A* is closer to *B* than *C* ($\Delta _{i,A} < \Delta _{i,C}$ for $i=1,2$). For this reason, both $a_1$ and $a_2$ express a preference for *A* over *C*. Since location *A* has $N_A = 2$ and one position is already taken, *A* must decide to accept either $a_1$ or $a_2$, depending on their fitness.

Agents, regardless of their age, will always try to relocate, however due to the higher probability to terminate their career older agents will eventually stop (see Entry and Exit). Conversely, younger agents move more because they are more likely to start out in lower fitness locations and thus will move more often until they reach a location from which they do not move away. In Fig. 3, we summarise and visualise the basic rules of our model. Moreover, in Fig. S19, we have a diagram presenting separately how we model and simulate locations and agents.

#### Matching agents to locations

In our model agents rank locations, while locations rank agents. To match locations and agents, we have to solve a matching problem similar to the stable marriage problem. However, our problem is slightly different as a location can accept more than one agent until the capacity $N_{K}(t)$ is reached. To solve this matching problem, we use an applicant proposing algorithm, similar to the NRMP-algorithm^34^. The details are given in the “Materials and methods” section.

#### Fitness dynamics

To model those agents not accepted at a new location, we consider that agent which stay at their current location, i.e., $r_i(t+1) = r_i(t)$, use the time step to further improve their fitness, $f_{i}(t)$. For this we assume a stochastic dynamics, precisely an additive stochastic process with a variance proportional to the fitness of the current location:

3 $$\begin{aligned} f_{i}(t + 1) = f_{i}(t) + \alpha _{\text {loc}} \eta, \end{aligned}$$

where $\alpha _{\text {loc}}$ is a parameter proportional to the quality of the agent location, and $\eta$ is a normally distributed stochastic variable with 0 mean and variance equal to 1 (i.e., $\eta \approx \mathcal {N}(0,1)$). By this, we assume that the change in fitness of an agent depends on its location. Also, it is not guaranteed that agents will improve their fitness; they can also lower it. At the end of each time step, we update the fitness of locations, $F_{K}(t)$, by averaging over the fitness $f_{i}(t)$ of all those agents that are currently based there. Hence, location and individual fitnesses are co-evolving in the model, but not analyzed in the present work.

#### Entry and exit of agents

At every time step, agents can exit and enter the system. This dynamic simulates the fact that academia is an open system, i.e., every year, scientists exit the systems, but also new ones enter it. Precisely, agents are removed with a small probability of $p_e$ at the end of every time step. Hence, the probability that an agents is removed from the simulation after *n* steps is $(1-p_e)^{n-1}p_e$. This process allows us to replicate the observed (academic) survival probability function of scientists (see Fig. S2 in SI).

#### Model calibration

We use the empirical data not only as an *input* to our model, to determine the *initial conditions*, but also for *calibration*. For this, we use only a subset of the available data (see the “Materials and methods” section). A major effort was spent to determine the optimal values of the two free parameters of our model, *b* and *s*. $b^{\text {opt}}=0.5$ means that a location *A* which is twice as far away as *B* must have a fitness $F_A=F_B \times \sqrt{2}$ to be as attractive as *B*. Moreover, $s^{\text {opt}}=0.5$ means that agents are accepted by locations with a probability larger than their fitness ratio. For example, an agent with fitness ratio $f_i/F_k=0.5$ is accepted with probability 0.71 and an agent with $f_i/F_k=0.25$ is accepted with $p = 0.5$.

#### Model validation

The calibrated agent-based model has to prove its evidence in that it can reproduce the whole set of empirical findings that have *not* been used during the calibration procedure. We *validate* the model by comparing two distributions on the level of scientists, and four distributions on the level of the mobility network. To simulate a large number of realisations, we focus on three neighbouring countries in Europe, namely Germany, France and the UK. Furthermore, we restrict the simulation to the period 2000 to 2006. The upper limit 2006 is given by the fact that the last publication in Author-ity is in 2009, and we require a 3-year window to identify moves. We do not use the period from 1950 to 2000 since they may represent academic systems from very different historical periods.

#### Results of agent-based simulations

**Figure 4:**
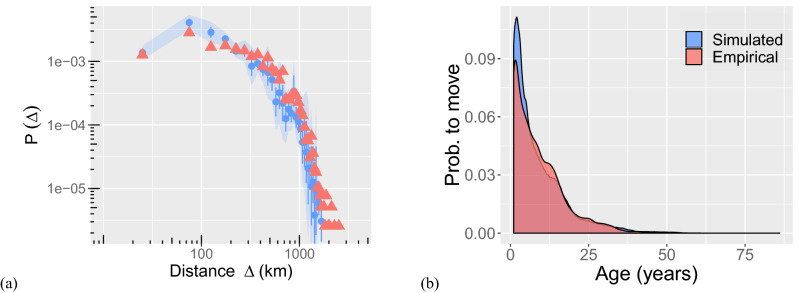
Comparison of simulated and empirical individual scientist features. (**a**) Distribution of relocation distances of scientists. (**b**) Distribution of moves dependent on the (academic) age of scientists, (red) indicates the empirical distribution, (blue) the distributions that are obtained from our agent-based simulations.

**Figure 5:**
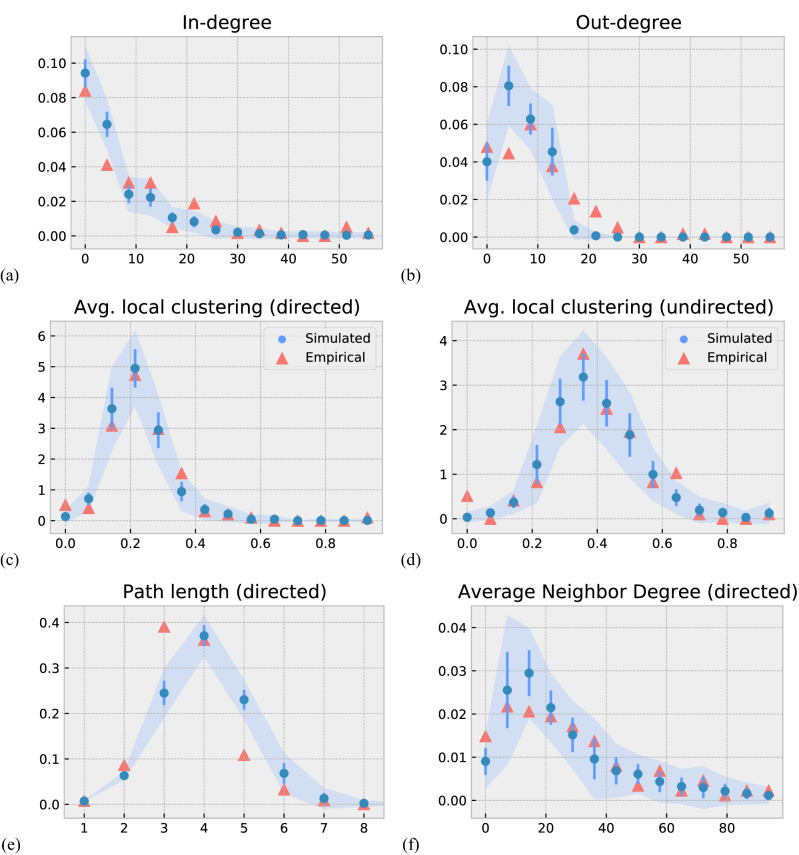
Comparison of empirical and simulated topological properties of the mobility network. Distributions of (**a**) in-degrees, (**b**) out-degrees, (**c**) local clustering coefficients (directed) (**d**) local clustering coefficients (undirected), (**e**) path lengths and (**f**) average neighbor in-degree. Red triangles indicates the empirical distribution, and blue circles distributions obtained from the simulation. The error bars correspond the standard deviations of 30 realisations of the simulation and the bands the 95% confidence interval.

The results of the validation are shown in Figs. 4 and 5. To allow for a direct comparison, we plot the empirical data in red and the simulation in blue. We can report a good match of all distributions both on the level of scientists and on the network level. Specifically, on the scientists’ level, we are able to reproduce the two distributions of *relocation distances* and of *age* when moving, see Fig. 4a,b.

On the network level, we are able to reproduce the four distributions of *in-degrees*, *out-degress*, *local clustering coefficients* and *average neighbor degree*, see Fig. 5a–f. We emphasise that these results are far from being trivial. As we start with an agent-based perspective, the results of our simulations refer to *career paths* of individual agents. From these, we have to reconstruct an aggregated network of mobility. Our simulation results for the network topology are reported for these simulated networks.

In conclusion, we report that our agent-based model captures the different features of the empirical data well, both on the scientists’ and the network level, without using direct information from these for the calibration.

## Discussion

This paper provides several results with relevance for both the empirical and the theoretical understanding of the global mobility of scientists. As a novel contribution, we introduce the concept of a *geographical career path* of an individual scientist, which can be extracted from data. Using records of 3.5 million scientists, we provide a statistical analysis of such career paths, that later form the basis for comparison with our model, on the scientists’ level. Aggregating over these career paths, we are further able to reconstruct the *world network of scientists’ mobility*, with cities as nodes and inflow/outflow of scientists as links. With this, we reveal the patterns of scientists’ mobility on two levels: the level of an individual scientist (age, relocation distance), and the level of cities forming a global network, which is a new empirical insight.

The most important contribution, however, is an agent-based model that allows reproducing these empirical findings on both the scientist and the city level. In our model, we assume as most relevant factors geographical distances, academic importance, and selectiveness of cities. The model uses as input for the initial conditions only variables that can be proxied by the available data. In particular, academic importance, denoted as the fitness of agents, is proxied from the available publications of scientists. The fitness of locations, another ingredient of the model, is then obtained by averaging over the fitness of agents at the particular location.

The agent-based model succeeds with simple assumptions for the relocation of agents. Agents rank all locations according to their fitness and their distance to the current location. However, they do not decide whether to move. This choice is made by the locations using the information on the fitness of the agents and capacity constraints. In essence, this poses a matching problem and can be related to similar problems discussed in the literature.

Our agent-based model only considers two free parameters, which need to be calibrated against the available data: *b* weights the spatial distance between the current location of an agent and any other location, *s* weights the selectiveness of locations when accepting agents that have a fitness below the location’s fitness. We find as *optimal parameters* $(s^{\text {opt}}, b^{\text {opt}}) = (0.5, 0.5)$. These parameters are maximally different from 0 or 1 and indicate that both selectiveness and distances are essential to reproduce the empirical mobility patterns. $b^{\text {opt}}=0.5$ characterizes the *supply side*, i.e., the ranking of locations by the *agents*. A location *A* needs to have $\sqrt{n}$ times the fitness of another location *B* if it is *n* times further away, to be equally attractive for an agent. $s^{\text {opt}}=0.5$ characterizes the *demand side*, i.e., the ranking of agents by *locations*. Provided there are sufficient openings available, an agent with a fitness ratio $f_i/F_k=0.5$ is accepted more than 2 out of 3 times, and an agent with $f_i/F_k=0.25$ still has a 50% chance to be accepted.

Let us emphasise once again that we find that both $s^{\text {opt}}$ and $b^{\text {opt}}$ are different from zero. This finding implies that both geographical distance and matching locations and agents’ fitness values are needed to reproduce the mobility network. When looking into simulations where $s=0$ and $b>0$, we find that the relocation distances $P(\Delta )$ is qualitatively reproduced, but the topological features are not (see Sect. 9 in the SI). This confirms the intuition that distance is a key ingredient to capture geographical aspects of scientist mobility, but it is not sufficient to capture the topology. Likewise, when looking into simulations where $s>0$ and $b=0$, only some topological properties are recovered, for example, the path length distribution (see Fig. S18e). However, other network features (such as the in- and out-degree) are not (see Fig. S18a,b). From a qualitative point of view, we find that disregarding prestige ($s=0$) has a larger effect on the model’s ability to capture the topology of the mobility network.

**Figure 6:**
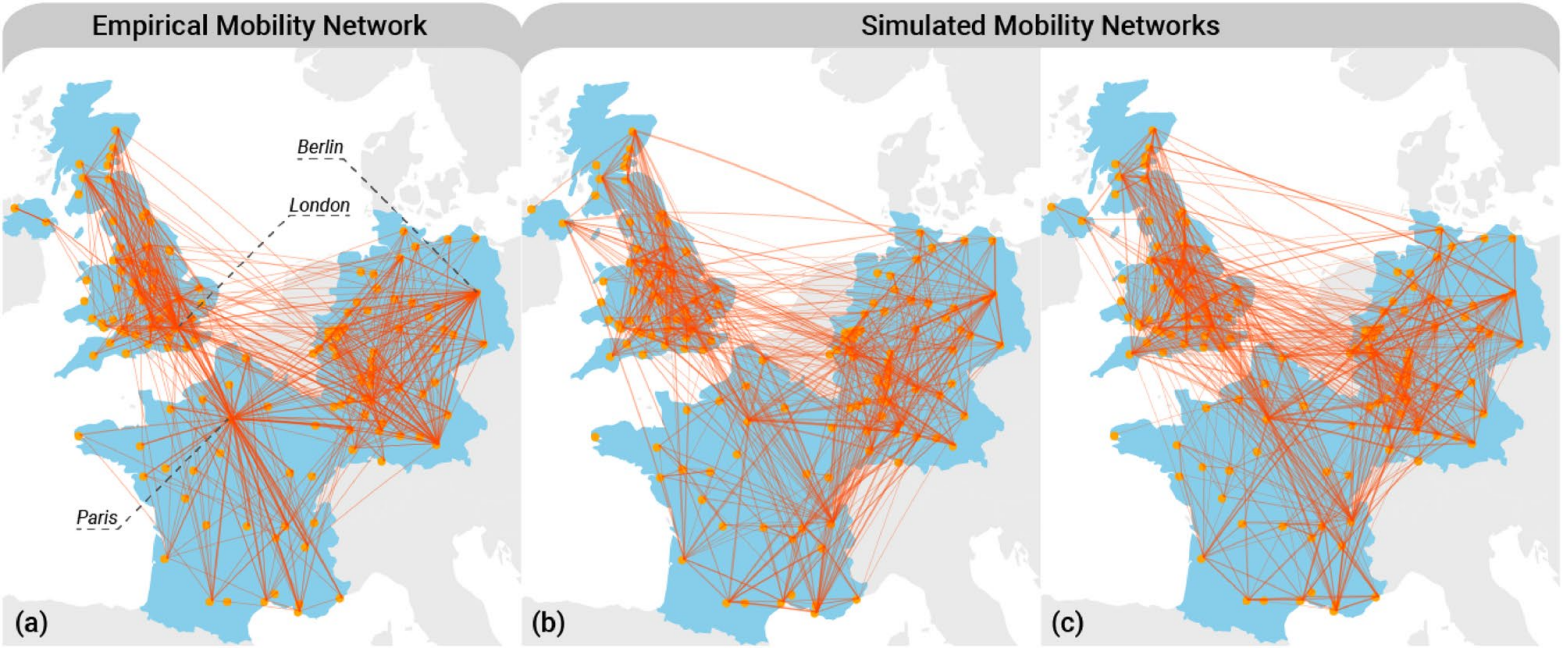
Empirical and Simulated mobility networks for France, Germany and UK. The empirical network (**a**) depicts the flows between cities as observed in the the period from 2000 to 2006. The thickness of the edges is proportional to the log of number of moves between the two cities. In (**b**,**c**) two realisations of the ABM are shown. In (**b**,**c**) 23,189 and 25,217 agents moving between 147 cities have been simulated, respectively. The difference in the number of agents is due to the stochastic entry and exit dynamics.

Using the model calibrated with the optimal parameters, our simulations match the available empirical data quite well. This is remarkable because the model does *not* include many factors which arguably play a role in the relocation decision. In other words, only using minimal assumptions about the supply (scientists) and demand (cities), and a simple matching mechanism, we are able to capture emergent features of the scientist mobility network. Some differences between the simulated and the empirical distributions become only noticeable if we plot the network of scientists’ mobility on the European scale, as shown in Fig. 6. We observe that the empirical network in Fig. 6a has more pronounced hubs than the simulated network shown in Fig. 6b,c. Moreover, our model predicts more exchanges between Germany and the UK, then we observe in the empirical network. Similarly, the model predicts more exchanges between less central German and British cities. Note also that in the empirical network, significantly more French cities are linked to Paris than among themselves compared to the simulations.

Finally, we stress that more factors are influencing the relocation choices of scientists than explicitly covered in our model. For example, quality of life, better networking opportunities or higher salaries might be relevant factors here. The more remarkable is the fact that our model, even at this level of detail, works considerably well.

In summary, we have provided the first agent-based model reproducing the mobility of scientists. In a data-driven approach, our model has been calibrated and validated against data, and we have found a remarkably good match between simulations and empirics. We show that minimal decision rules capture many complex features of the observed mobility of scientists. Besides, we have quantified the relative importance between geographical distances and academic attractiveness from the perspective of a scientist trying to relocate.

## Materials and methods

### Extracting individual career paths of scientists

For our work, we use the MEDLINE database, the largest open-access bibliographic database in the life sciences. Our analysis is based on two datasets provided by Torvik and co-authors, namely MapAffil^35^ and Author-ity^36^, which have been extracted from MEDLINE. MapAffil lists for each MEDLINE paper and each scientist the *disambiguated city names* of the listed affiliation (37,396,671 city-name instances). It further gives a unique identifier as well as the geo-coordinates of each city. Author-ity contains the *disambiguated scientist names*, linking them to their respective publications. Combining these two sources of information about geo-coordinates and time allows us to construct the geographical “career path” of scientists, using the approach of^16^.

Formally we denote a career path of a scientist $i \in N$ as a sequence $p_i$, for example, $p_i=\{A_{t_0}$, $A_{t_1}$, $A_{t_2}$, $B_{t_2}$, $B_{t_4}$, $C_{t_5}$, $C_{t_6}\}$. *A* denotes the city as defined by its geo-location $R_{A}=(X,Y)$ where *X* gives the latitude and *Y* the longitude according to the data from MapAffil. The subscript $t_{0}$ refers to the year scientist *i* was affiliated in the respective city, according to the career path data obtained. For more information about the data used, see the SI.

### Determining locations in geographical space

Defining the boundaries of a city is a central problem in urban studies. A standard definition available for US cities is the “Metropolitan Statistical Area” (MSAs)^37^. However, as the name suggests, this definition is *not* available outside the US. Therefore, to identify cities, we rely on the definition of “location” provided by Google Maps. This definition reflects administrative boundaries, which are not perfect substitutes. As argued by^38,39^ natural and administrative definitions follow different size distributions. However, because we do not argue about the size distribution of cities, this is not a crucial concern.

### Determining the free model parameters

Parameter *b* weights the impact of spatial distances on the individual rankings of agents. $b=0$ would imply that distances do not play a role in relocation preferences; with $b=1$, a location *A* which is twice as far away as location *B* needs to have twice the fitness of *B* ($F_A=2\times F_B$) to be equally attractive. In general, a location *A* which is *n* times as far away as *B* must have a fitness $F_A = n^{b} \times F_B$ to be as attractive as *B*.

Parameter *s* weights the flexibility of locations to still accept agents with a fitness less than the fitness of the location. For $s=0$, the probability of a location to accept agents is independent of their fitness and always equal to 1 ($p(K,i)=1$); with $s=1$ the probabilty to be accepted is equal to the ratio between the agent’s and location’s fitness ($f_i/F_k$), e.g., an agent with $f_i= 0.5 \times F_k$ will be accepted with probability 1/2. In the case of $s>1$, an agent is accepted by a location with a probability smaller than their fitness ratio ($p(K, i) < f_i/F_k$).

### Calibration procedure

To *calibrate* the model parameters *b*, *s*, we use an established approach in agent-based modeling^28^, machine learning^40–42^ and computer simulations in general^43^. It combines two elements: (a) a grid search and (b) a performance score.

The grid search consists of an exploration of the (low dimensional) parameter space through computer simulations. For *b* and *s* we consider the values $\{0.0$, 0.005, 0.01, 0.05, 0.1, 0.5, 1.0, 5.0, $10.0\}$.

For each parameter combination, we obtain from the simulations two distributions for the *inflow* and *outflow*. To determine the optimal combination of (*b*, *s*), we compare the simulated and the empirical inflow and outflow distributions. For this comparison, we use a performance score based on the Kolmogorov–Smirnov (KS) statistic^44^.

Precisely, for each combination of parameters (*b*, *s*), we compute the KS-statistic between the empirical and simulated distributions of inflow, $D_1(b,s)$, and of outflow $D_2(b,s)$. We then define the performance score as $1 / ( D_1(b,s) \times D_2(b,s) )$, such that the optimal combination $(b^{\text {opt}}, s^{\text {opt}})$ maximizes this score.

From the calibration procedure, we find *optimal parameters* $(s^{\text {opt}}, b^{\text {opt}}) = (0.5, 0.5)$. The comparison between the empirical and the simulated distributions is shown in Fig. S7a,b in SI. The close match demonstrates that our model is correctly calibrated.

Note that we use the intercity distance as an input for the model, and the *b* parameter weights its importance. However, we do not use distance information during the calibration procedure. We use instead information on the inflow and outflow of scientists (i.e., a “stock” summary statistics). Hence, if it is not trivial, whether the model is able to reproduce geographical aspect of scientist mobility.

### Simulation initialisation

At the beginning of the simulation, we populate the cities at only 80% capacity, which means that we initiated the simulation with 22,000 agents, and ca. 300 locations. As the starting year $t=0$, we take 2000. From each city, we take its geographical position and the number of scientists in the year 2000. We assign these quantities to locations to characterize their $R_{K}$ and $N_{K}(t=0)$. The initial fitness value of a location, $F_{K}(t=0)$, is determined by averaging over the fitness values of those agents based in the given city in 2000.

From each scientist, we obtain its geographical position (in a given city), his/her academic impact, and the years of activity as of the year 2000. We assign these quantities to agents to characterize their $r_{i}(t=0)$, $f_{i}(t=0)$ and $y_{i}(t=0)$. The academic impact is proxied by the papers that a scientist has authored in the two years prior. As above described, we assign to each paper a score equal to the impact factor of the journal where it was published divided by the number of co-authors. Then, for each scientist, we sum the scores of the papers he/she has co-authored between 1998 and 2000. This defines the starting fitness of agents, i.e., $f_i(t=0)$. We then run the agent-based model using parallel updates of all agents per time step.

### Simulating the entry and exit dynamics of agents

Our empirical analysis finds that the number of scientists is almost constant in 6 years time windows (see Fig. S3 in SI). Thus, the total number of agents is almost constant during our simulations. Specifically, the number of new agents $n_n$ is proportional to the number of removed agents $n_r$ at the previous time step. We sample $n_n$ from a Gaussian distribution with mean $n_r$ and standard deviation $\sigma = n_r (0.1/2)$.

### Simulating the matching problem

To match agents with locations, we first create a ranking of the agents according to their fitness. Starting from the agent with higher fitness, we look at its top five preferred locations. If one of these locations accept the agent, we move it there. When an agent *i* has moved to a new location *K*, we update its position vector, $r_i(t+1) = R_{K}$, and keep its fitness constant, $f_i(t+1) = f_i(t)$. Then, we consider the second agent in the ranking and keep trying to match it to a new location. With this approach, we ensure that agents relocate to their preferred locations if they are accepted. Also, since we first try to match agents with higher fitness, locations obtain agents with higher fitness, i.e., their preferred ones.

## Supplementary Information

Supplementary Information.

## Data Availability

The raw XML data on all MEDLINE articles are available for download from the NIH at https://www.nlm.nih.gov/databases/download/pubmed_medline.html, ftp://ftp.ncbi.nlm.nih.gov/pubmed/baseline The disambiguation of authors (Authority) and affiliations (MapAffil) has been obtained from http://abel.lis.illinois.edu/downloads.html Access to this resource can be requested for free from the maintainers through the online form on the same page. Note that due to an agreement with the providers of Authority and MapAffil, these datasets may only be shared by requesting access through the previously mentioned online form. We make the aggregated mobility network at city level available with no individual identifying information through figshare after publication.
