# Supplementary material for: Reproducing scientists’ mobility: a data-driven model
Source: Sci Rep. 2021 May 24;11:10733. doi: 10.1038/s41598-021-90281-9 (PMC8144224; doi:10.1038/s41598-021-90281-9)
Supplement: Supplementary file 1 — Supplementary Information. [file 41598_2021_90281_MOESM1_ESM.pdf]

# Supplementary Information for: Reproducing scientists' mobility: A data-driven model

**Giacomo Vaccario**[1], **Luca Verginer**[1,2,*], **Frank Schweitzer**[1]

[1] *ETH Zürich, Chair of Systems Design, Department of Management, Technology and Economics, Weinbergstrasse 56/58, CH-8092 Zürich, Switzerland*
[2] *IMT School for Advanced Studies Lucca, AXES Lab, piazza S. Francesco 19, IT-55100 Lucca, Italy*

[*] *Corresponding author: fschweitzer@ethz.ch*

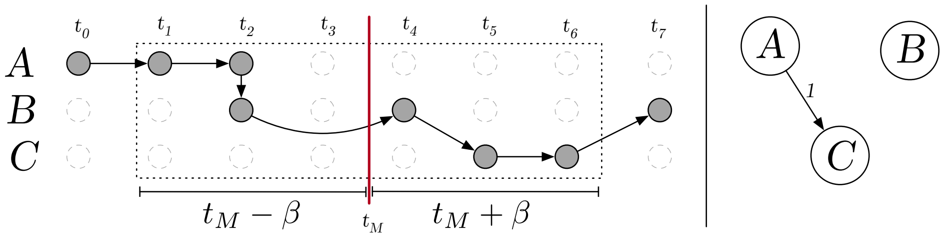


Figure S1: Illustration of procedure to extract movements

## 1 Data

We use two datasets extracted from MEDLINE, which is the largest open access bibliographic dataset in the life science [1]. The first dataset is `Author-ity` Torvik and Smalheiser (2009) that contains *disambiguated scientist names* and links them to their respective publications in MEDLINE up to 2009. The second dataset is `MapAffil` Torvik (2015) which lists for each scientist the *disambiguated city names* of their affiliation (37 396 671 city-name instances) listed in the MEDLINE publications up to 2015. Note that `MapAffil` covers MEDLINE up to 2015, and `Author-ity` covers MEDLINE up to 2009. This discrepancy means that we can only use the years up to 2009 when combining the datasets.

By combining the two datasets, we extract for each scientist his/her "career path". An example of such a career path is shown in Table S1. The merged dataset contains a total of of $N = 3\,740\,187$ career paths of scientists, which were active between 1950 and 2009, traversing $M = 5\,485$ unique cities.

Table S1: Example of career path of a specific scientist (LM Shul.). Only a subset of his/her publications is shown. For each record we have the year of publication, the city of the affiliation and the relative PubMed ID identifying the paper.

| Year | City | Pubmed ID |
|---|---|---|
| ⋮ | ⋮ | ⋮ |
| 2000 | Miami, FL, USA | 11054153 |
| 2000 | Miami, FL, USA | 10928576 |
| 2000 | Miami, FL, USA | 10714670 |
| 2000 | Miami, FL, USA | 10634252 |
| 2001 | Baltimore, MD, USA | 11763581 |
| 2001 | Baltimore, MD, USA | 11391746 |
| 2002 | Baltimore, MD, USA | 15177058 |
| ⋮ | ⋮ | ⋮ |

We have a time resolution of one year. During a full year, a scientist has often multiple publications, and sometimes is located in multiple cities. In Figure S1, we illustrate this case as we observe locations $A$ and $B$ simultaneously during year $t_2$. When this happens, we choose the location that has been observed

[1] https://www.nlm.nih.gov/bsd/pmresources.html

more often in that year. If two locations are equally frequent, then we select the one that is closest to the move year $t_M$. In case of perfect ties, one location is chosen at random. Our procedure follows the approach of Verginer and Riccaboni (2018).

## 2 Entry and exit dynamics

**Exit probability.** We assume that a scientist has left academia if he/she does not publish for two years in a row. By counting the number of scientists leaving academia by academic age, we obtain the empirical survival probability (and its complement, the exit probability). Precisely, in Figure S2 we show the Complementary Cumulative Distribution Function (CCDF) for the scientists leaving academia by academic age. Note that this CCDF is well matched by an exponential function with $\lambda \approx 0.1$. This finding motivate us to chose the exit probability to $p_e = 0.1$ in our simulations.

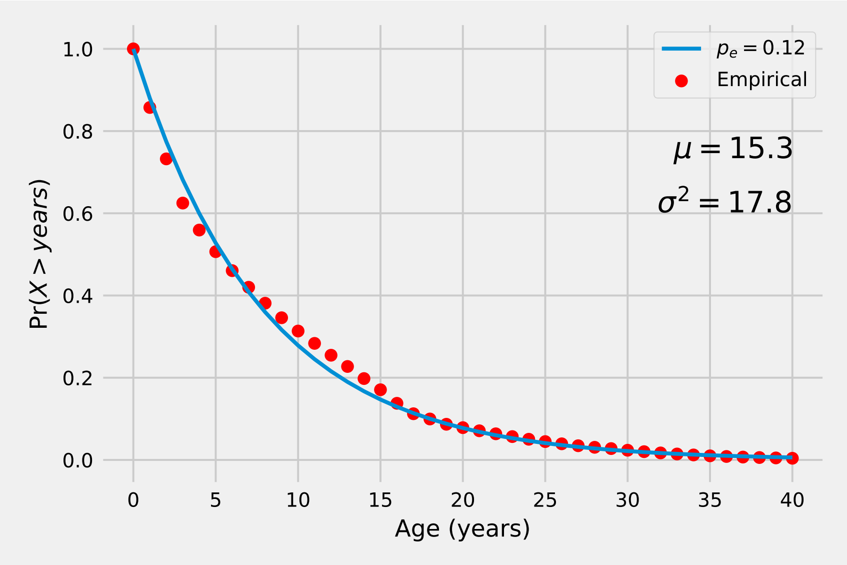


Figure S2: Career Path CCDF. The Complementary Cumulative Distribution shows the probability for scientists to remain active, i.e., to publish at least one paper after $k$ years. The red markers represent the the empirical CCDF. The continuos blue line is the fitted CCDF using an exponential distribution.

**New agents.** In Figure S3, we report the number of scientists per city in France, Germany, and the UK between 2000 and 2004 (i.e., the countries and period used to calibrate and validate the model). This number is almost constant, albeit with a slight positive growth trend In Figure 4 we show the growth rate in the years 2002 and 2004 relative to 2000, highlighting again, that size while increasing, does so slowly (at least in the the period chosen). Given the near stationarity of city sizes over the chosen period we do not model city growth explicitly. Tackling a city's scientist population growth is left to future refinement. For these reasons, we assume that the number of new agents $n_n$ is close to the number of

removed agents $n_r$. Moreover, we allow for some small fluctuations by sampling $n_n$ from a gaussian distribution with mean $n_r$ and standard deviation $\sigma = n_r(0.1/2)$.

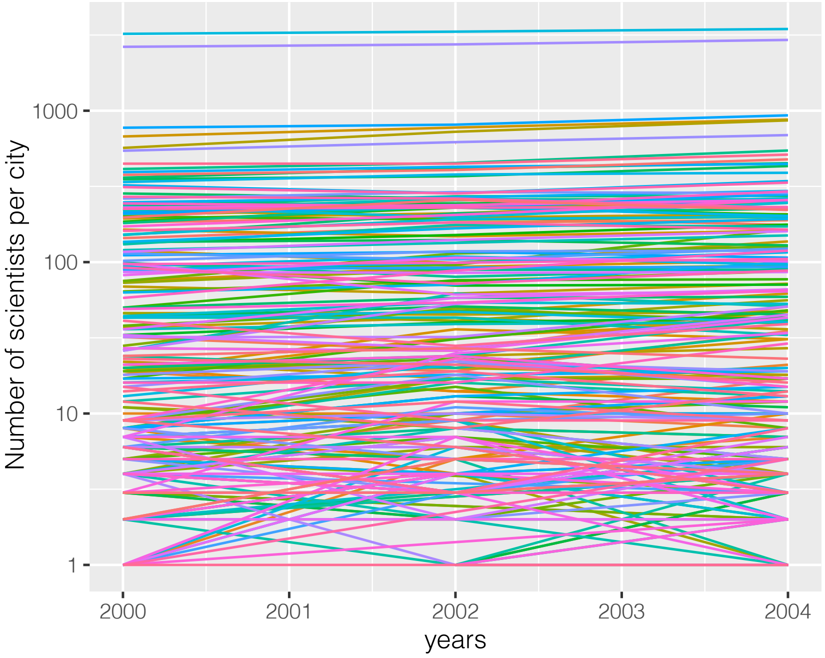


Figure S3: The number of scientists per city in function of time. Each line is the number of scientists per city. We use a log scale in order visualize the heterogeneity of city sizes.

## 3 Summary of the data-driven modeling

In Figure S5, we summarize our modeling procedure. We start from analyzing the data from which we obtain a set of macroscopic observations at the scientist and city level. These observations range from the academic productivity of scientists to city inflows and outflows of scientists. Then, we divide these observations in three disjoint sets. A first set contains observations that act as input data. Precisely, we use this first set for defining the microscopic rules, the parameters, and the initialization of the model. We report the observations used as input data in Table S2 and Table S3.

A second set of observations is used to calibrate the model. In Table S4, we report this second set together with the key ingredients of the calibration procedure. The details of this procedure are in Sect.4. In a nutshell, we obtain the best parameters for the model by comparing simulations with empirical observations. We call the *calibrated model* a *data-driven model* as both the input variables (e.g., initial conditions, agent and location features) and the parameters are obtained from data.

Finally, the third set of observations is used to validate the calibrated model (see Table S5). In the validation procedure, we check whether the model reproduces macroscopic observations both at *scientist*

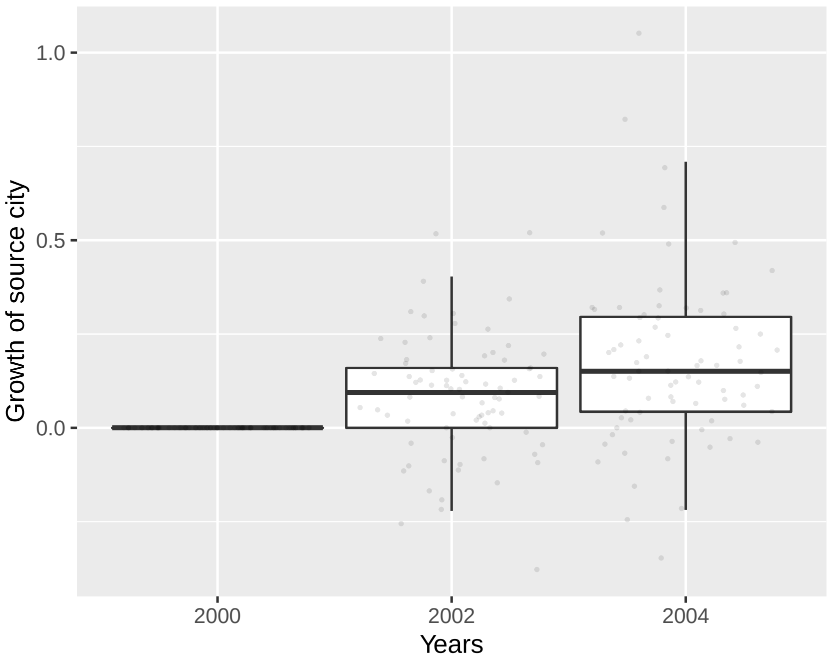


Figure S4: Growth of city size relative to 2000, computed as $\text{Size}_t/\text{Size}_{2000}$

| **Locations** | |
|---|---|
| **Input data** | **Model feature** |
| Number of scientists in cities | Carrying capacity of locations |
| Academic impact of scientists aggregated over city | Starting masses of locations |
| Geographical positions of cities | Positions of locations |
| Almost constant city sizes in 5 years time windows | Constant carrying capacities |

Table S2: Data used as input together with their respective model features for locations.

and *network* level. These observations are the distributions of moved distances, Figure 1 (a), the "age at move" distributions, Figure 1 (b), and four distributions of the topological feature shown in Figure 1(c-f).

We note that this is quite an ambitious goal since our model needs to reproduce several *dissimilar* system dimensions (i.e., scientists and intercity) correctly. If the model can reproduce the described distributions, we have a strong indication that the interaction rules governing scientist and city interactions capture a relevant aspect of the real mobility of scientists. The information available to the model during calibration does not imply the more complex validation measures. If we find that the simulated results agree with the empirical validation metrics, it means that the *interaction rules* are the reason for the observed patterns and good validation results.

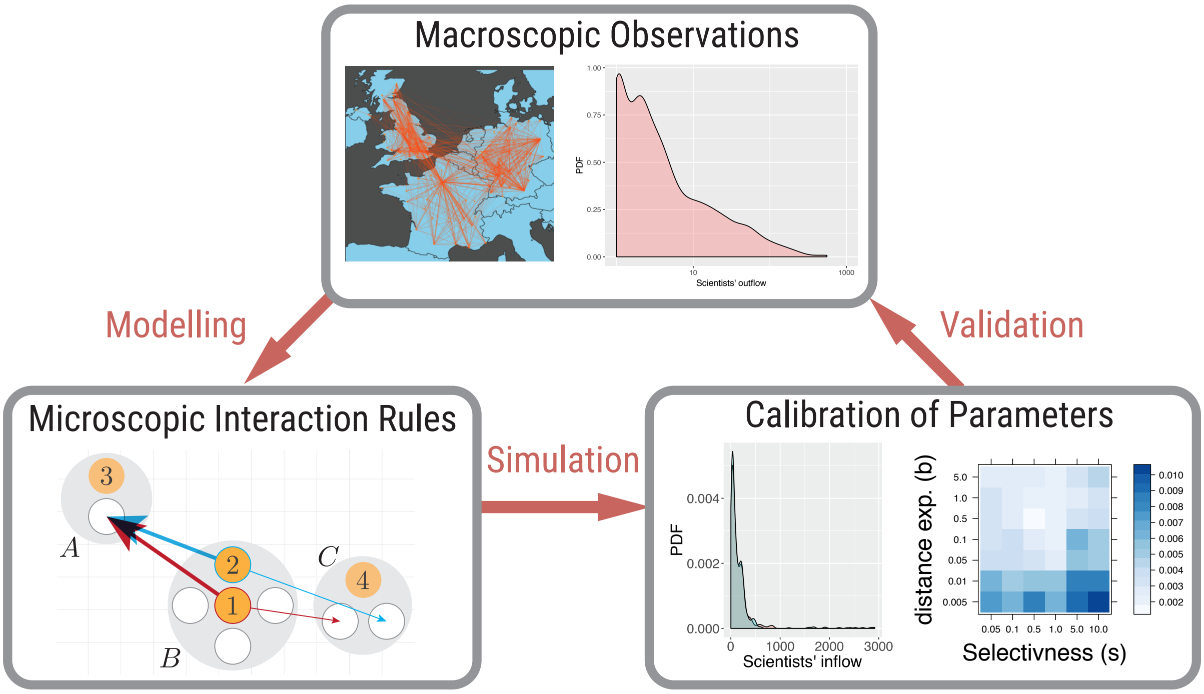


Figure S5: Our modeling procedure is divided in three parts. First, we collect and analyze data about scientists and their career paths across cities. From these data, we extract a set of macroscopic observations both at the scientist and city level (top panel). Second, we use the macroscopic observations to define and inform the model that should reproduce scientists' mobility (bottom left panel). Once we have the model, we simulate and calibrate it using real-data (bottom right panel). We call the calibrated model a data-driven model. Finally, we validate the data-driven model by comparing simulation results with the macroscopic observations.

| **Agents** | |
|---|---|
| **Input data** | **Model feature** |
| Academic impact of scientists | Starting fitness of agents |
| Academic age of scientists | Starting academic age of agents |
| CCDF of scientists leaving academia | Exit of agents at a rate $\lambda$ |
| Almost constant city sizes in 5 years time windows | New agents $\approx$ removed agents |

Table S3: Data used as input together with their respective model features for agents.

## 4 The calibration procedure

In the calibration procedure, we use a set of empirical observation that will not be used for the validation. In particular, we choose the distribution of in- and outflow of scientists (see Figure S6). As written in the main manuscript, we define for each year $t$ the number of scientists $\Delta N_{K\leftarrow L}(t)$ moving into city $K$ from another city $L$, i.e., the inflow, and the number of scientists $\Delta N_{L\leftarrow K}(t)$ moving out of city $K$ to another city $L$, i.e. the outflow.

| **Distributions used for calibration** | |
|---|---|
| Network-level | PDF of scientists' inflow (Figure S6 (a))<br>PDF of scientists' outflow (Figure S6 (b)) |
| **Grid search of the parameter space** | |
| $b$ | $\{0.005, 0.01, 0.05, 0.1, 0.5, 1.0, 5.0\}$ |
| $s$ | $\{0.05, 0.1, 0.5, 1.0, 5.0, 10.0\}$ |
| **Performance score** | |
| $\frac{1}{N}\sum_k^N D_1(b,s) \times D_2(b,s)$ | $D_1$, KS-statistic between the distributions of inflow<br>$D_2$, KS-statistic between the distributions of outflow<br>$N$, number of simulations per combination of parameters |

Table S4: Summary of the key ingredients of the calibration procedure. We report the distribution used to calibrate the model, the parameter space explored during the grid search, and the performance score used to evaluate the goodness of parameter combinations.

| **Reproduced distributions** | |
|---|---|
| Network | PDF of degree (Fig. 5 (a-b))<br>PDF of clustering coefficients (Fig. 5 (c) and (d))<br>PDF of shortest path lengths (Fig. 5 (e))<br>PDF of av. neighbour degree (Fig. 5 (f)) |
| Scientists-centric | PDF to observe a scientists to move given his age (Fig. 4 (a))<br>PDF of distances travelled by scientists (Fig. 4 (b)) |

Table S5: Summary of the distributions used for validation. Note that there macroscopic observations both at the scientist and city/network level.

Figure S6 (a) and (b) show the respective distributions for the aggregated inflow $\Delta N_K^{\text{in}}(t) = \sum_L \Delta N_{K \leftarrow L}(t)$ of scientists into city $K$ and the aggregated outflow $\Delta N_K^{\text{out}}(t) = \sum_L \Delta N_{L \leftarrow K}$ of scientists

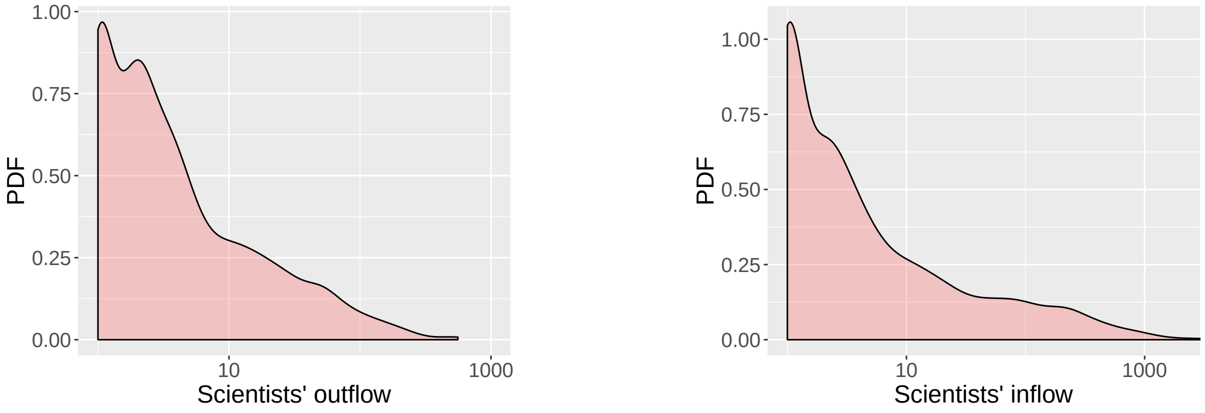


(a) (b)

Figure S6: Distributions of (a) inflow of scientists into any city, (b) outflow of scientists out of any city. The $x$-axis is in log-scale.

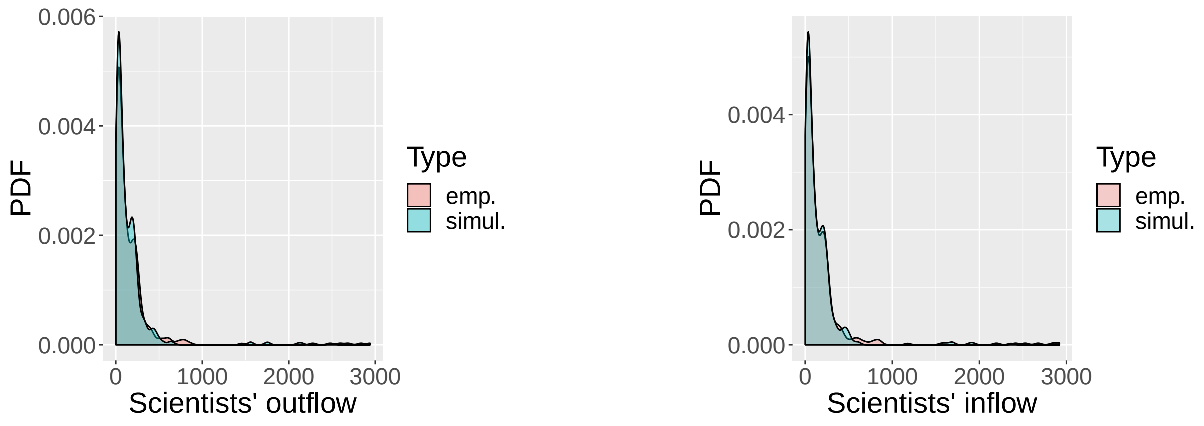


(a) (b)

Figure S7: Distributions of (a) inflow of scientists into any city, (b) outflow of scientists out of any city. (red) indicates the empirical distributions, (blue) the (optimally) simulated distributions obtained from the calibration of our agent-based model.

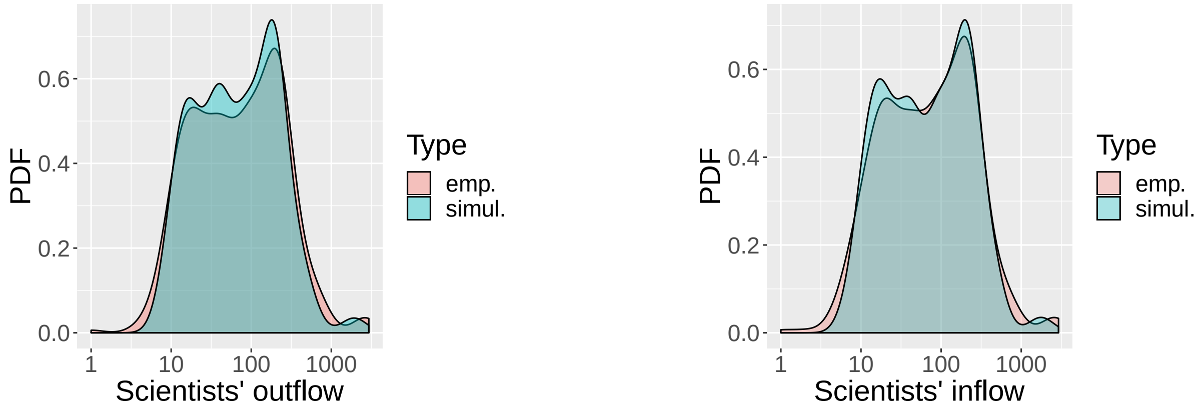


(a) (b)

Figure S8: Distributions of (a) inflow of scientists into any city, (b) outflow of scientists out of any city. (red) indicates the empirical distributions, (blue) the (optimally) simulated distributions obtained from the calibration of our agent-based model. The $x$-axis is in log-scale.

out of city $K$. The aggregate in- and the out-flow are computed at three different time windows centred at 2000, 2002 and 2004. This means that each city is considered three times (once in each time window). We note the left-skew distribution for both quantities, which indicates the *heterogeneous* contribution of cities to the global movement of scientists.

To calibrate the model, we use the two empirical distributions: the inflow and the outflow distributions shown in Figure S7(a,b). Note that, we calibrate our model considering only cities and scientists present in three countries: France, Germany and United Kingdom with at least 10 scientists. We remove cities with fewer scientists as the computation of a city fitness is reliable only when having a certain

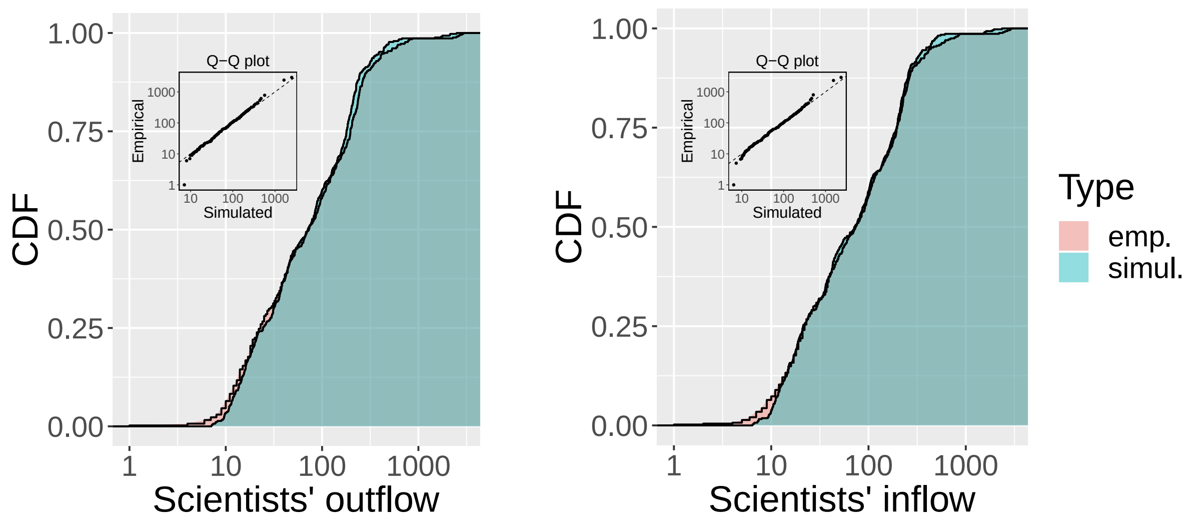


(a) (b)

Figure S9: Cumulative distributions of (a) inflow of scientists into any city, (b) outflow of scientists out of any city. (red) indicates the empirical distributions, (blue) the (optimally) simulated distributions obtained from the calibration of our agent-based model. The $x$-axis is in log-scale. In the inset, we also report the Q-Q plot for the two samples.

amount of scientists. For this reason, Figure S6 and Figure S8 differ.

In the calibration procedure, we start by performing a grid search, i.e., we explore the parameter space defined by our two parameters $b$ and $s$. For both $b$ and $s$, we consider the values $\{0.0, 0.005, 0.01, 0.05, 0.1, 0.5, 1.0, 5.0, 10.0\}$. Note that with these values of $s$, we explore very different shapes of the acceptance probability $p(K, i)$ defined in the main text. In Figure S10, we plot the acceptance probability $p(K, i)$ for five different values of the parameter $s$. Note that for $s = 10$, locations becomes more selective as almost only agent with $f_i \geq F_K$ are accepted. For smaller values of $s$, locations becomes less selective. For example, for $s = 0.05$, locations accept agents almost independently of their fitness. In Figure S10, we plot the acceptance probability $p(K, i)$ for five different values of the parameter $s$ and is given by

$$p(K, i) = \begin{cases} 1 & \frac{f_i}{F_K} \geq 1 \\ \left(\frac{f_i}{F_K}\right)^s & \text{otherwise} \end{cases}$$

Note that at large values of $s = 10$, locations becomes more selective and accept almost exclusively agents with $f_i \geq F_K$, i.e. only agents which will increase a city's fitness $F_K$ For smaller values of $s$, locations becomes less selective. For example, at $s = 0.05$, locations accept agents almost independently of their fitness. Likewise, we change $b$ in a range of parameters that imply that distance play no role $b = 0$, to $b = 10$ for which distance dominates any agent preference. For example, given two locations one with fitness $F_K$ at a distance $d$ and one with fitness $F_L = 50F_K$ at a distance $1.5d$, an agent prefers the closer location, $K$. In other words, for $b = 0$, a 50% increase in the distance cannot be compensated

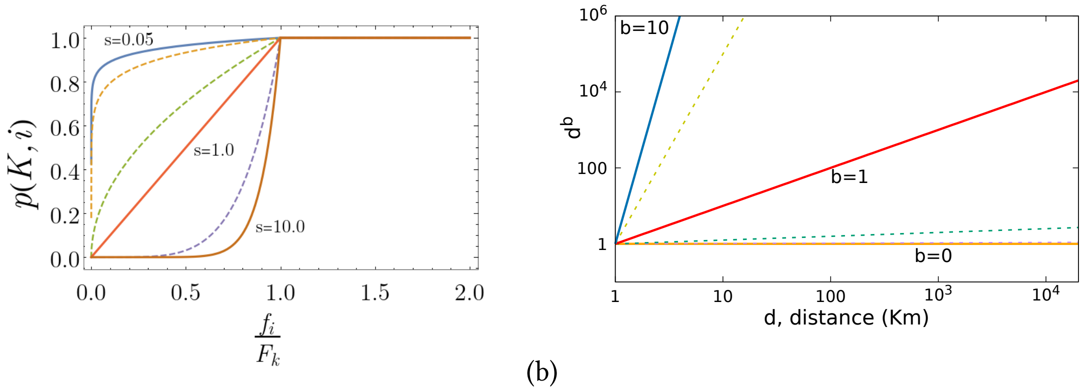


Figure S10: (a) Probability of a location $K$ to accept an agent $i$ in function of the ratio between the agent and location fitness. We set $s = 0.05, 0.5, 1, 5$, and 10 respectively in blue, yellow, green, red, purple, and orange. (b) The effect of changing $b$: for $b = 0$ (orange), 0.01 (purple), 0.1 (green), 1 (red), 5 (yelow), 10 (blue). For small $b$ changes in the distance do not affect agents preferences, while for large $b$ further locations becomes extremely less preferred.

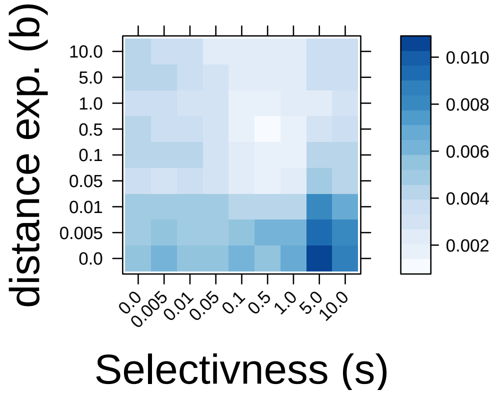


Figure S11: The heat-map shows the results of the grid-search on the two parameters $s$ and $b$. The color of each cell corresponds to a $p$ for a given $(b, s)$ pair as described in eq. 1. The optimal parameter pair $(b^{opt}, s^{opt})$ is $(0.5, 0.5)$.

with fitness more than 50times bigger.

To decide which parameter combination better reproduces the in- and outflow distributions, we use the following performance score:

$$p^{opt} = (b^{opt}, s^{opt}) = \arg\min_{b,s} \frac{1}{N} \sum_{k}^{N} D_1(b, s) \times D_2(b, s) \tag{1}$$

where $N$ is the number of simulations, $D_1(b, s)$ is the Kolomogorov-Smirnov statistic between the empirical and simulated distributions of city outflow. $D_2(b, s)$ is the Kolomogorov-Smirnov statistic between the empirical and simulated distributions of city inflow. In oder words, the performance score for each combination of parameter is the average product between $D_1(b, s)$ and $D_2(b, s)$. The optimal combination of parameter is the one that minimizes this score.

In Figure S11, we report a heat-map showing the exploration of the parameter space. For each combination of parameters, we have performed $N = 10$ simulations. We find as *optimal parameters* $p^{opt} = (s^{opt}, b^{opt}) = (0.5, 0.5)$. This means that both selectiveness and distances better reproduce the empirical data when they give a sub-linear contribution.

## 5 The Verification

In addition to the plots in the main text highlighting the good match between the empirical and simulated results, we show here in addition their qq-plots along with the p-value of the relative KS test. The KS test has as null hypothesis that the two distributions are the same. This means that for a low p-value the two distributions are different and for high (e.g. above 0.05 they are similar). We see that for all tests we have a p-value above 0.10 suggesting again a good match between the simulated and empirical distributions. Note that the KS test has been carried out on the bins also depicted in the validation figures.

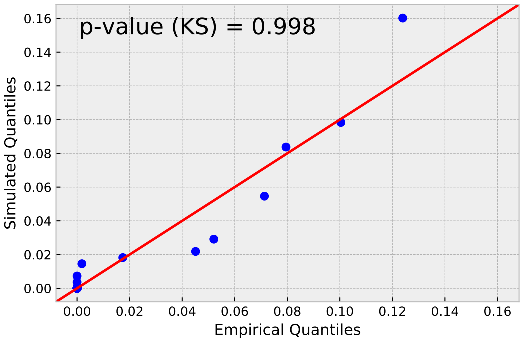


Figure S12: QQ Plot of empirical vs simulated Average Neighbor In-Degree

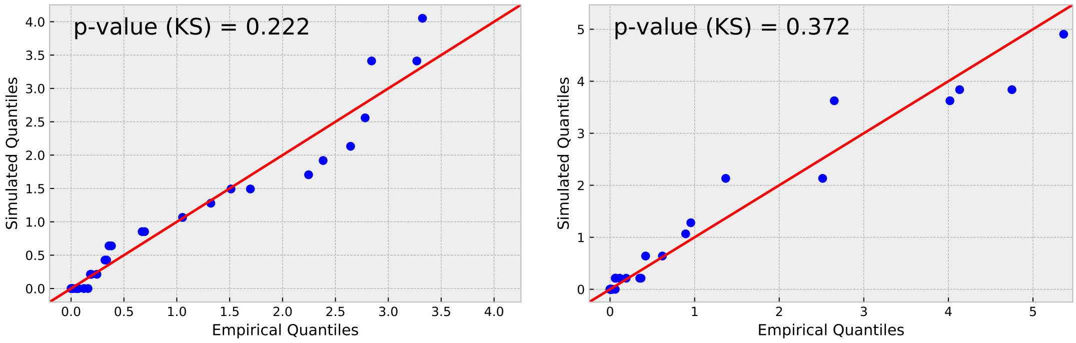


Figure S13: QQ Plot of empirical vs simulated for (left) directed local clustering coefficient and (right) undirected local clustering coefficient.

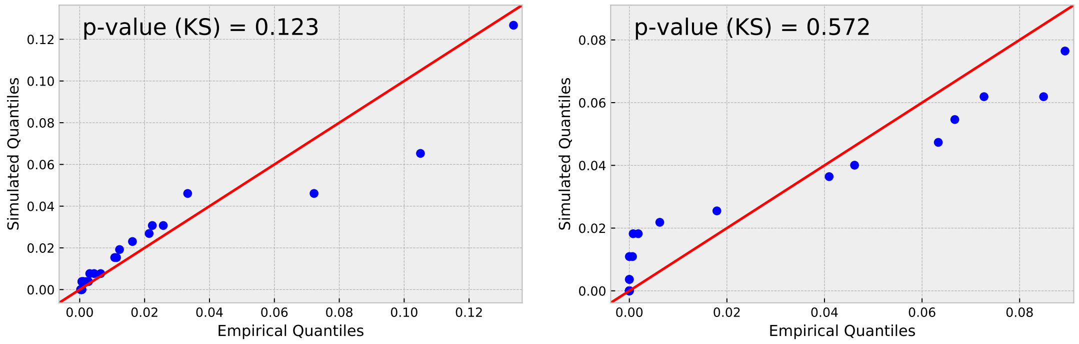


## 6 Assortativity Measures

| Degree source | Degree target | not weighted | weighted |
|---|---|---|---|
| in | in | -0.1067 | -0.096 |
| | out | -0.1000 | -0.0889 |
| out | in | -0.1163 | -0.1041 |
| | out | -0.1088 | -0.0972 |

Table S6: Assortativity for various network representations

In Table 6, we report the average assortativity mixing with 8 permutation of the directed assortativity measure: ‘in-in’, ‘in-out’, ‘out-out’, ‘out-in’ for both weighted and binary representations. We find that the assortativity pattern is neutral (or slightly negative) for all the different permutations. For this analysis we have used the assortativity measure of Newman (2003) implemented in `networkX` (Hagberg *et al.*, 2008) Also, we have looked into the average degree connectivity defined in Barrat *et al.* (2004) and implemented in `networkX`. From visual inspection, it is easy to confirm that the assortativity is quite neutral, and for the unweighted (i.e., binary) network it is slightly more negative.

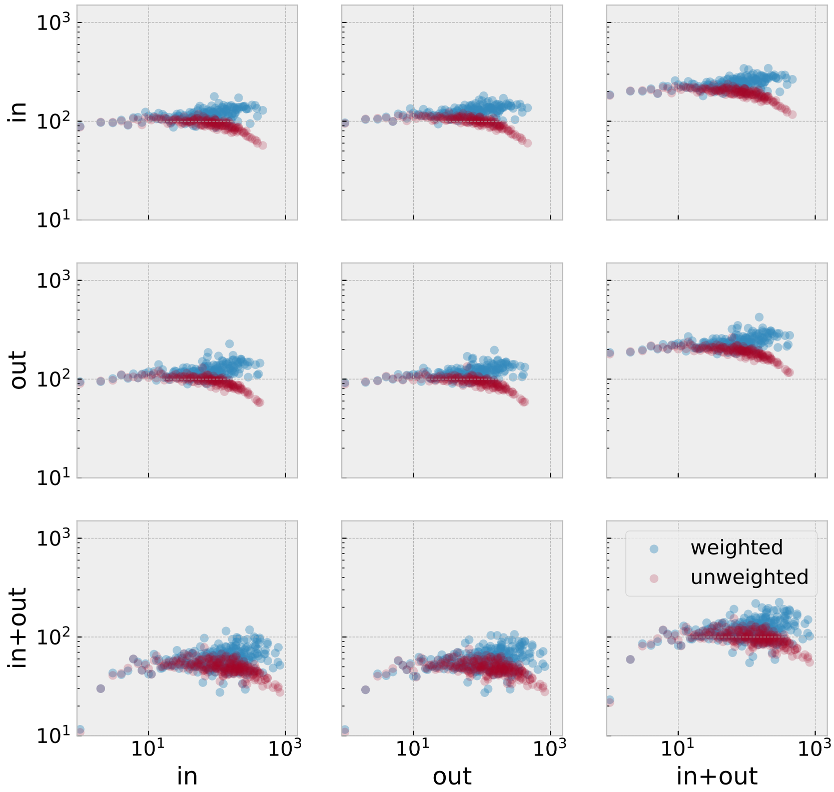


Figure S14: Average degree assortativity for 18 possible permutations: 'in-in', 'in-out', 'in-(in+out)', 'out-out', 'out-in', 'out-(in+out)', '(in+out)-in', '(in+out)-out', '(in+out)-(in+out)' for both the weighted and binary mobility networks.

## 7 Correlation analysis of city fitness

In Figure 15 and 15, we plot the correlation between fitness of city and other quantities. We do find a some correlation only between the longitudinal position of the cities and their fitness. This correlation is mainly generated from the presence of many cities with high fitness in the U.S.

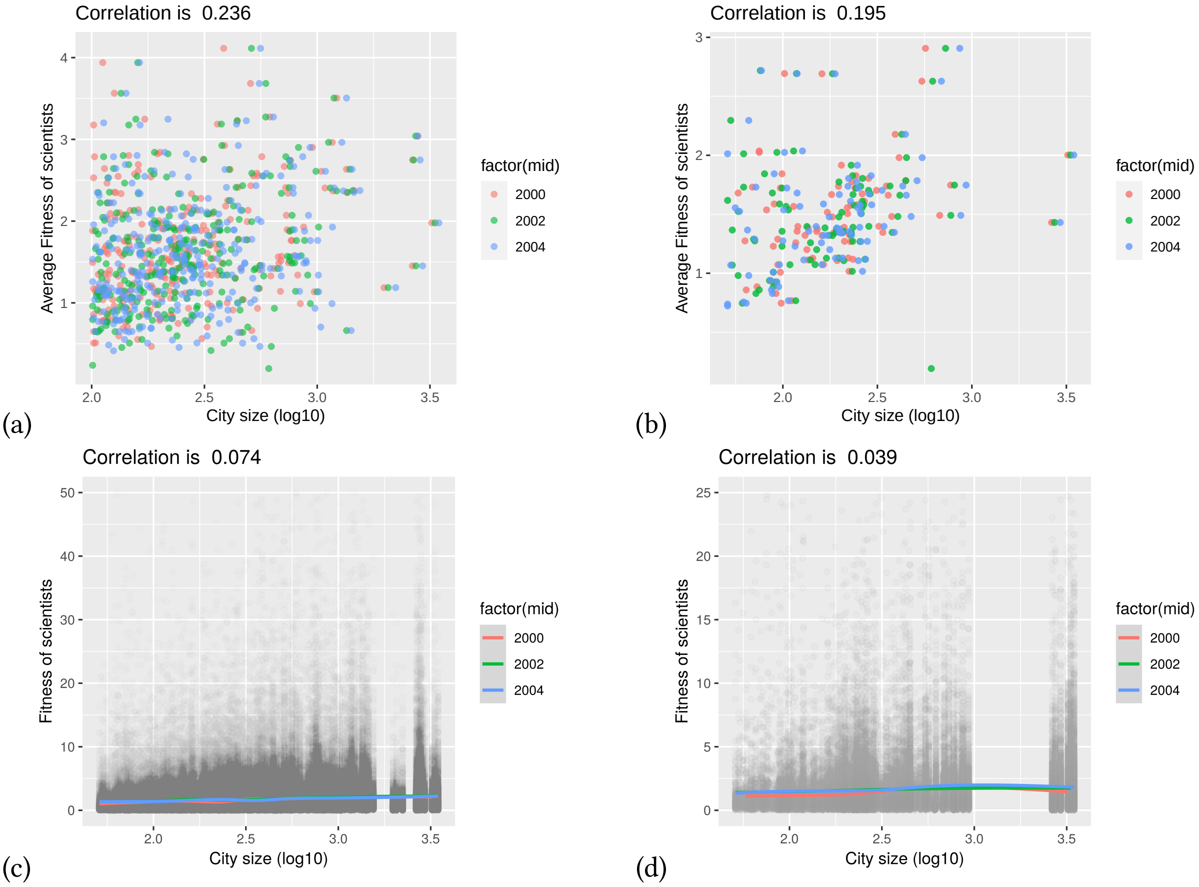


Figure S15: Correlation plots between fitness of city and other quantities for the entire data (left) and only for cities belongings to UK, France and Germany (right). In (a,b) we report the correlation between city fitness and city size. In (c,d) we report the correlation between scientist fitness and city size.

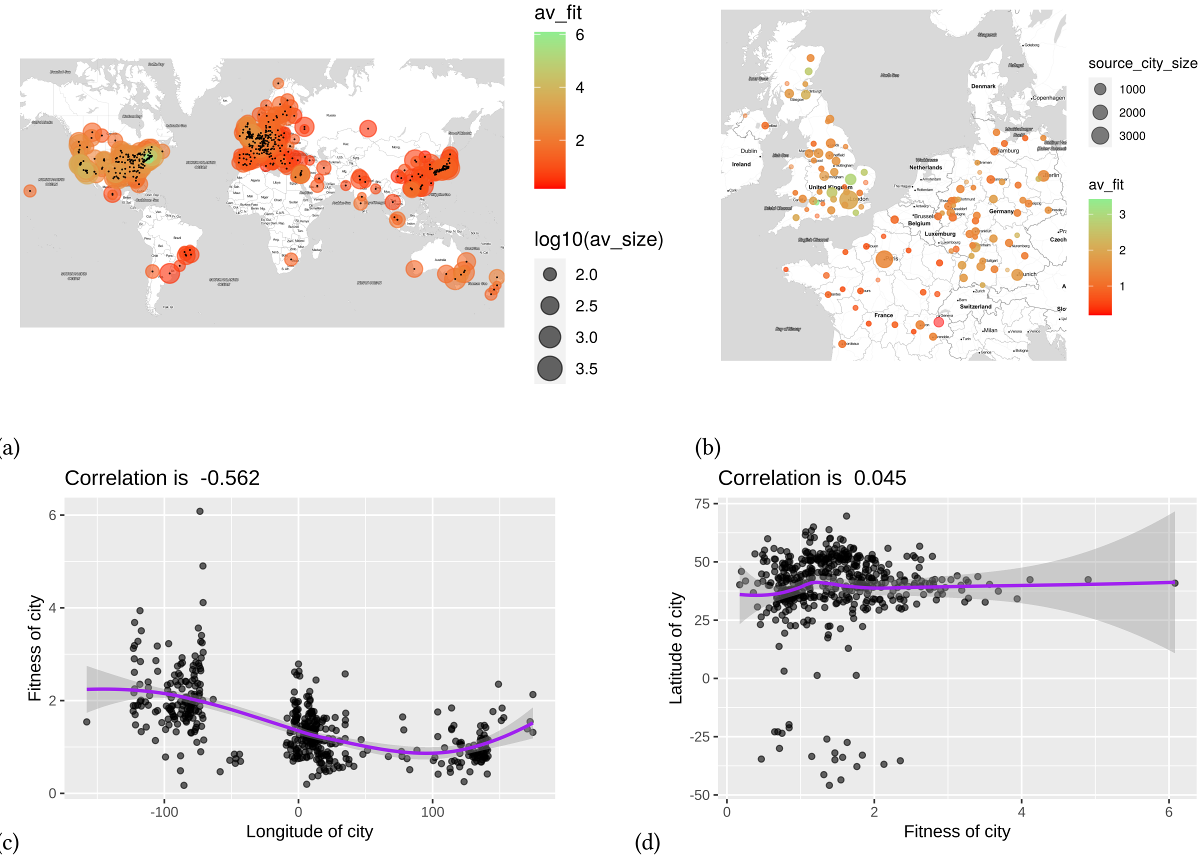


Figure S16: In (a,b) we visualize the geographical distribution of city fitness (green high fitness, red low fitness) and city size distribution (using the size of circles). In (c) we report the correlation between city fitness and their longitude. In (d) we report the correlation between city fitness and their latitude.

## 8 Comparison with the configuration model

We now compare our model with a random base-case model obtained using a configuration model. Precisely, to better understand the role of fitness and distance, we now produce a null model that removes their effect. We use the inflow and outflow of cities to define their in and out-degree. Then, we rewire the cities randomly, i.e., we generate networks using a weighted (multi-edged, to be precise) and directed configuration model. In such a model, both distance and fitness of the city do not play a role. The networks generated from this model have by construction the same in and out-degree distribution as the empirical network.

When looking at the average path length of the generated networks by the configuration model, we find that they underestimate the empirical mean (see Table7). We argue that this underestimation comes from the fact that configuration model has no information on intercity distance, and hence, connections between faraway locations are more comma than expected in the empirical network. This mechanism introduces “shortcuts” in the networks and reduces the average path length. Also, when looking at the average clustering coefficient of the networks generated with the configuration model, we find that they overestimate the empirical mean (see Table7). For example, when two scientists move from smaller peripheral cities (e.g., one in France and another in Germany) to a larger city (e.g., London), the probability to observe a move between the peripheral cities is small. In the configuration model, the different attractiveness (i.e., fitness) is lost, and the city size (i.e., the degree) is not sufficient to recover this property. Finally, note that our “motivated” model has the opposite behaviour when estimating the average path length and clustering coefficient. Precisely, our model overestimates the average path length and slightly underestimates the average clustering coefficient. These results strongly indicate that by introducing distance and fitness, we obtain a “motivated” model very different from a random one.

| | Av. Clustering | | Av. path length | |
|---|---|---|---|---|
| | mean $\mu$ | std $\sigma$ | mean $\mu$ | std $\sigma$ |
| Emprical | 0.27 | — | 2.62 | — |
| Optimal | 0.26 | 0.01 | 3.03 | 0.12 |
| DW-Configuration | 0.42 | 0.01 | 2.08 | 0.01 |

Table S7: We report the average clustering coefficient and path length for the empirical mobility network, and for the networks generated with the optimal model and with a directed and multi-edged configuration model. Note that for the empirical network the average value does not have standard deviation as we have one single observation. For both models, we have computed the standard deviations using 30 network realizations.

## 9 Exploration of the impact of the selectiveness and distance

**The absence of selectiveness.** In Fig.17, we report the comparison between the simulation with ($s$=0.0, $b$=0.5) adn the empirical data. By setting $s = 0$, locations accepts any agents that tries to move there if they have enough capacity. With this rule, we have that agents' fitness does not play a role in the acceptance mechanism. This fact implies that the agents will always move and be accepted in the city with higher fitness. This phenomenon makes the out-degree distribution more narrow see Fig. 17(b) and compare it also to Fig. 5(b) in the manuscript). Moreover, we observe that the both clustering distribution are poorly matched, and in general the network simulated have smaller average clustering coefficient. We hypothesize that this mismatch derives from the fact that scientists move more easily between institutions with similar prestige. Evidence of institutional constraints restricting scientists mobility have been investigated in (Vaccario *et al.*, 2020). Studying and confirming the above hypothesis represents an interesting avenue for future research.

**The absence of distance..** We find that removing distance has a qualitatively smaller negative effect in reproducing the topology of the network (see Fig. 18). At the same time, we find that the locations in the simulated network have a higher out-degree (see Fig. 18(b)). To understand this, recall that when setting $b = 0$, agents move to any locations with higher fitness without considering their distance. Hence, the set of "interesting" locations becomes larger, and more out-going links are created by those agents accepted in the locations. This type of process increases the out-degree of the locations compared to the case with $b > 0$ (compare Fig. 18(b) with (Fig. 17(b) and Fig. 5(b)).

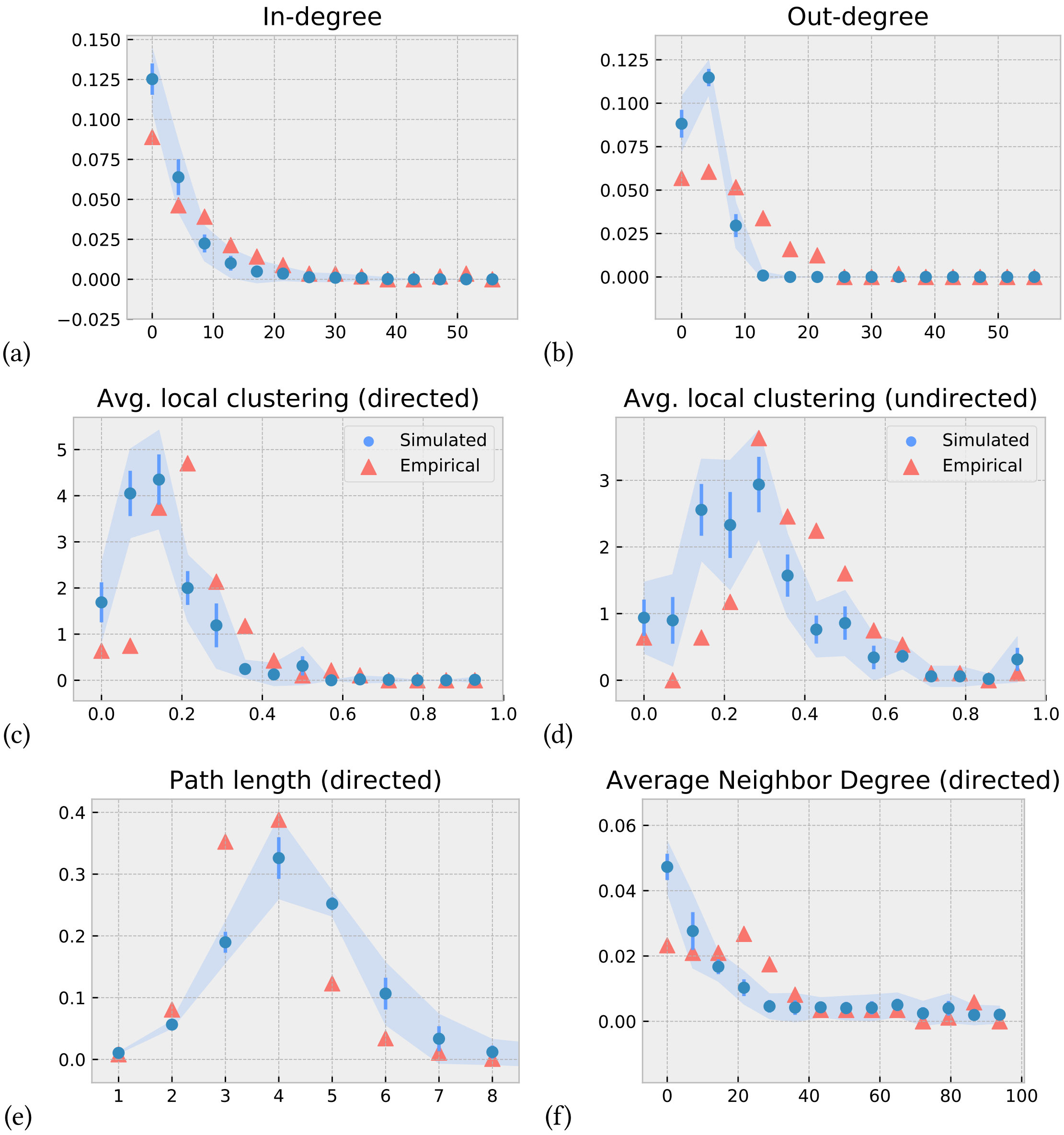


Figure S17: *Comparison of empirical and simulated-(s=0.0, b=0.5) topological properties of the mobility network.* Distributions of (a) in-degrees, (b) out-degrees, (c) local clustering coefficients (directed) (d) local clustering coefficients (undirected), (e) path lengths and (f) average neighbor in-degree. Red triangles indicates the empirical distribution, and blue circles distributions obtained from the simulation. The error bars correspond the standard deviations of 30 realisations of the simulation and the bands the 95% confidence interval.

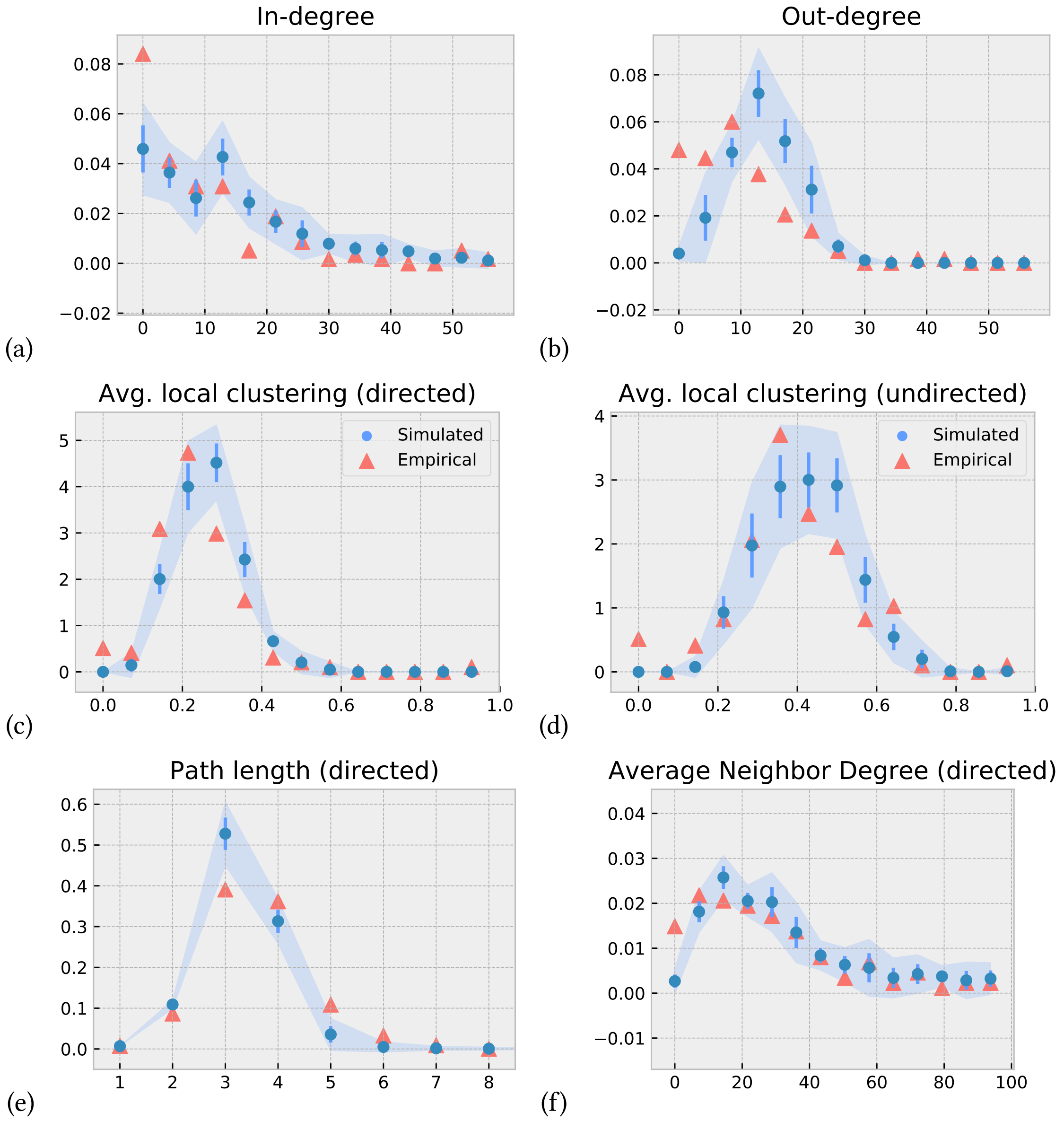


Figure S18: *Comparison of empirical and simulated-(s=0.5, b=0.0) topological properties of the mobility network.* Distributions of (a) in-degrees, (b) out-degrees, (c) local clustering coefficients (directed) (d) local clustering coefficients (undirected), (e) path lengths and (f) average neighbor in-degree. Red triangles indicates the empirical distribution, and blue circles distributions obtained from the simulation. The error bars correspond the standard deviations of 30 realisations of the simulation and the bands the 95% confidence interval.

## 10 Diagrammatic presentation of the simulated model

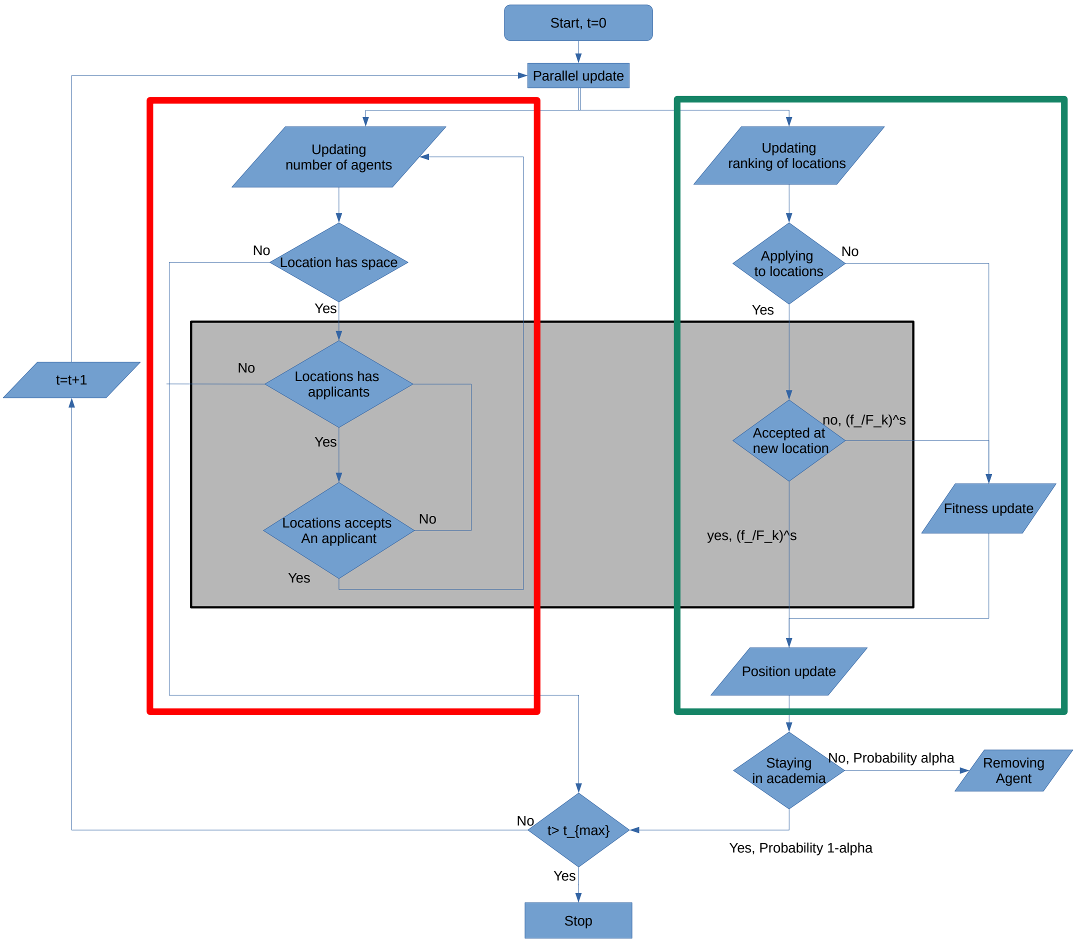


Figure S19: Diagrammatic representation of the simulated model. The diagram has to be read from top to bottom. The red box contains the location dynamic, while the green box the agent dynamic. The black transparent box represents when the matching algorithm acts to organize the agents and location interactions.

## References

Barrat, A.; Barthelemy, M.; Pastor-Satorras, R.; Vespignani, A. (2004). The architecture of complex weighted networks. *Proceedings of the national academy of sciences* **101(11)**, 3747–3752.

Hagberg, A. A.; Schult, D. A.; Swart, P. J. (2008). Exploring Network Structure, Dynamics, and Function using NetworkX. In: G. Varoquaux; T. Vaught; J. Millman (eds.), *Proceedings of the 7th Python in Science Conference*. Pasadena, CA USA, pp. 11 – 15.

Newman, M. E. (2003). Mixing patterns in networks. *Physical review E* **67(2)**, 026126.

Torvik, V. I. (2015). MapAffil: A Bibliographic Tool for Mapping Author Affiliation Strings to Cities and Their Geocodes Worldwide. *D-Lib Magazine* **21(11/12)**.

Torvik, V. I.; Smalheiser, N. R. (2009). Author name disambiguation in MEDLINE. *ACM Transactions on Knowledge Discovery from Data* **3(3)**, 1–29.

Vaccario, G.; Verginer, L.; Schweitzer, F. (2020). The mobility network of scientists: Analyzing temporal correlations in scientific careers. *Applied Network Science* **5(1)**, 36.

Verginer, L.; Riccaboni, M. (2018). Brain–Circulation Network: The Global Mobility of the Life Scientists. *(Working Papers)* **(10/2018)**.
